# Functional Brain Changes During Mindfulness-Based Cognitive Therapy Associated With Tinnitus Severity

**DOI:** 10.3389/fnins.2019.00747

**Published:** 2019-07-24

**Authors:** Benjamin Zimmerman, Megan Finnegan, Subhadeep Paul, Sara Schmidt, Yihsin Tai, Kelly Roth, Yuguo Chen, Fatima T. Husain

**Affiliations:** ^1^Department of Speech and Hearing Science, University of Illinois at Urbana–Champaign, Champaign, IL, United States; ^2^Beckman Institute for Advanced Science and Technology, University of Illinois at Urbana–Champaign, Champaign, IL, United States; ^3^Neuroscience Program, University of Illinois at Urbana–Champaign, Champaign, IL, United States; ^4^Department of Statistics, The Ohio State University, Columbus, OH, United States; ^5^Department of Statistics, University of Illinois at Urbana–Champaign, Champaign, IL, United States

**Keywords:** tinnitus, mindfulness-based cognitive therapy, resting state MRI, functional MRI, graph connectivity analysis

## Abstract

Mindfulness-based therapies have been introduced as a treatment option to reduce the psychological severity of tinnitus, a currently incurable chronic condition. This pilot study of twelve subjects with chronic tinnitus investigates the relationship between measures of both task-based and resting state functional magnetic resonance imaging (fMRI) and measures of tinnitus severity, assessed with the Tinnitus Functional Index (TFI). MRI was measured at three time points: before, after, and at follow-up of an 8-week long mindfulness-based cognitive therapy intervention. During the task-based fMRI with affective sounds, no significant changes were observed between sessions, nor was the activation to emotionally salient compared to neutral stimuli significantly predictive of TFI. Significant results were found using resting state fMRI. There were significant decreases in functional connectivity among the default mode network, cingulo-opercular network, and amygdala across the intervention, but no differences were seen in connectivity with seeds in the dorsal attention network (DAN) or fronto-parietal network and the rest of the brain. Further, only resting state connectivity between the brain and the amygdala, DAN, and fronto-parietal network significantly predicted TFI. These results point to a mostly differentiated landscape of functional brain measures related to tinnitus severity on one hand and mindfulness-based therapy on the other. However, overlapping results of decreased amygdala connectivity with parietal areas and the negative correlation between amygdala-parietal connectivity and TFI is suggestive of a brain imaging marker of successful treatment.

## Introduction

Subjective tinnitus, the perception of sound in the absence of an external source, is a currently incurable chronic condition with a relatively large prevalence rate (12–30% of the general population) (McCormack et al., 2016). Tinnitus is a heterogeneous condition with varying perceptual qualities (e.g., pitch, loudness, type of sound) as well as varying psychological reactions to the condition. Most individuals habituate to the condition over time, but for a subset of people, the condition becomes debilitating with severe psychological effects including comorbid anxiety and depression (Bartels et al., 2008; McCormack et al., 2015).

The pursuit of better understanding and treating the condition typically falls into two overlapping lines of research. One area seeks to understand and intervene on the primary perception of the sound. The other line of research focuses more on understanding the reasons for the variability in psychological severity of the condition and pursuing ways to effectively encourage habituation and management of the perception.

Recently, mindfulness-based therapies have been introduced as a treatment option in this second category of research to reduce the psychological burden of the condition. Although the research on these treatments are still in their early days, mindfulness-based therapies have shown initial promise in modulating the severity of tinnitus (Kreuzer et al., 2012; Philippot et al., 2012; Gans et al., 2014; Roland et al., 2015; McKenna et al., 2017). These therapies teach a conception of mindfulness defined by Kabat-Zinn (1994) as ‘paying attention…on purpose, in the present moment, and non-judgmentally.’

As mindfulness-based therapies have grown in popularity and have shown efficacy in a number of psychological conditions (e.g., Hofmann et al., 2010; Philippot et al., 2012; Hölzel et al., 2013; Lakhan and Schofield, 2013), there has been a growing body of literature investigating changes in functional brain activity after a period of mindfulness training. Research on the functional correlates of tinnitus has also expanded in recent years, with evidence pointing to differences in both responses to certain types of tasks as well as resting state functional activity predictive of the condition. Given the evidence for functional changes with tinnitus and functional changes due to mindfulness training, there has been growing interest in testing the hypothesis that the positive effects from mindfulness interventions on tinnitus severity are modulated through changes in brain function (Roland et al., 2015). Previous work on functional connectivity changes with tinnitus have suggested that therapies which increase connectivity with limbic regions and attention resting state networks could be effective at reducing tinnitus-related distress (Schmidt et al., 2013, 2017). Since mindfulness-based interventions have observed this pattern of connectivity change (Tang et al., 2015), there is a strong theoretical basis to expect that mindfulness-based therapies may be effective through this mechanism. In this study, it was hypothesized that mindfulness-based therapies alleviate symptoms of tinnitus by altering those functional patterns.

In addition to changes in seed-voxel connectivity, supplemental tools from network analysis are widely used to understand anatomical and functional brain connectivity patterns using data obtained from neuroimaging studies (Bassett and Bullmore, 2006; Bullmore and Sporns, 2009; Rubinov and Sporns, 2010; Meunier et al., 2010; Hutchison et al., 2013; Sporns, 2014; Zalesky et al., 2014; van den Heuvel et al., 2016; Bassett and Sporns, 2017). Various network metrics have been used to assess and differentiate between individuals in terms of cognitive abilities and disease progression in a wide range of conditions (Liu et al., 2008; Alexander-Bloch et al., 2010; Lynall et al., 2010; van den Heuvel et al., 2010, 2013; Bassett et al., 2011; Yu et al., 2011; Hutchison et al., 2013; Wang et al., 2013; Braun et al., 2015). Researchers have also observed many salient properties of brain functional networks, e.g., community (module) structure, rich club organization, network hub structure, strong local clustering, short characteristic path length, etc., which together lead to efficiency and small world property in networks. These network properties, in turn, have been associated with efficient performance of brain’s tasks. A number of studies have also applied graph analysis tools to data from mindfulness or meditation studies and have found significant differences in efficiency and clustering coefficient in functional brain networks to be associated with meditation (Gard et al., 2014; van Lutterveld et al., 2017). However, these graph analysis tools have not been applied with respect to changes in tinnitus outcomes across mindfulness-based interventions.

In the current pilot study, we build upon results presented in Husain et al. (2019) showing a reduction in tinnitus severity after a mindfulness-based cognitive therapy (MBCT) intervention by analyzing the functional MRI (fMRI) data in the same subjects. We analyzed changes in brain activity during an auditory emotion task and during resting state across three sessions before, immediately after treatment, and 8 weeks after the treatment ended. The auditory emotion task was used in previous studies (Carpenter-Thompson et al., 2014) to differentiate between bothersome and mild tinnitus. As a secondary goal, we investigated whether changes in functional activity to the auditory emotion task or resting state across the sessions predicted the reductions in tinnitus severity, as assessed through the Tinnitus Functional Index (TFI: Meikle et al., 2012). Finally, we investigated how network properties change across the mindfulness intervention and if those changes reflect tinnitus severity. This study adds to the burgeoning literature on functional brain differences in effective tinnitus therapies and will deepen our knowledge of this complex and heterogeneous condition.

## Materials and Methods

### Participants

#### Inclusion/Exclusions Criteria

Participants underwent a screening session to determine eligibility before enrolling in the study. Individuals included in the study were between the ages of 21 and 72, had a Tinnitus Handicap Inventory (THI: Newman et al., 1996) score of 28 or greater and had pure-tone thresholds less than or equal to 30 dB HL up to 2 kHz testing frequency in order to ensure that they were able to hear the MBCT instructors. The goal of recruitment was to include a broad sample of individuals with chronic, bothersome tinnitus, while excluding comorbid symptoms that may too drastically increase the heterogeneity of the tinnitus condition investigated in the study or otherwise impair the ability of the subject to participate in the study. These included transmandibular joint problems, a history of Meniere’s disease, pulsatile tinnitus, drug and/or alcohol abuse in the recent past, a score greater than 25 on the Beck Anxiety Inventory (BAI: Beck et al., 1988a), a score greater than 30 on the Beck Depression Inventory (BDI: Beck et al., 1988b) or a score greater than 0 on question 9 of the BDI, which asks about suicidal ideation, neurological disorders (e.g., epilepsy), a history of or currently unmanaged post-traumatic stress disorder, unmanaged chronic health problems (e.g., hypertension, diabetes), a score of 23 or below on the Mini-Mental State Examination (MMSE: Folstein et al., 1975), or speech discrimination scores in quiet lower than 80% in the better ear.

#### Sample Demographics

Twenty-one participants (11 female) with problematic tinnitus were recruited from the Champaign–Urbana area via fliers, campus notifications, and newspaper advertisements. The study was approved by the University of Illinois at Urbana–Champaign Institutional Review Board (IRB Protocol Number: 16784), and all subjects gave written informed consent prior to taking part in each phase (audiological, MRI, intervention) of the study.

Of those 21 participants, 12 participants participated in the initial MRI scanning session, 10 participants remained for the post-MBCT session, and 8 participants returned for a follow-up scan 8 weeks after the conclusion of the study. Demographics for the 12 imaged participants are included in Table 1. Supplementary Table 1 shows the extended demographics for the post-MBCT and follow-up sessions.

**TABLE 1:** Sample demographics.

**Gender**	**M = 5; F = 7**			
	**Mean**	***SD***		
Age (year)	51.42	10.63		
TFI	50.92	15.57		
BDI	6.67	7.04		
BAI	10.17	8.67		

**Mean hearing thresholds at screening**				

**Frequency (kHz)**	**Right ear mean threshold (dB HL)**	**Right ear threshold SD (dB HL)**	**Left ear mean threshold (dB HL)**	**Left ear threshold SD (dB HL)**

0.25	12.08	5.42	15.00	5.22
0.5	12.08	5.82	12.92	6.56
1	14.17	7.93	14.58	7.82
2	16.67	6.51	15.42	11.37
3	25.42	13.22	25.42	16.44
4	26.25	15.69	25.42	16.16
6	35.42	20.17	30.42	17.38
8	34.17	22.04	37.08	21.05
9	51.25	20.79	43.33	20.60
10	53.75	23.27	46.67	22.50
11.2	56.67	23.77	51.25	25.86
12.5	59.58	25.89	56.25	27.64
14	64.58	24.16	60.83	26.01
16	50.83	17.69	47.08	20.28

### Mindfulness-Based Cognitive Therapy

Participants were enrolled in one of two 8-week MBCT sessions. The classes were taught by clinical psychology graduate students trained in delivery of MBCT and supervised by a licensed clinical psychologist. Classes were structured according to the MBCT curriculum developed by Segal et al. (2002) and used the student workbook developed for MBCT by Teasdale et al. (2014). Each class lasted about 2 h and involved learning about mindfulness, participating in exercises designed to illustrate principles of mindfulness, clarifying values and setting goals, and practicing techniques designed to cultivate mindfulness. After each class, home practice assignments were given according to the workbook, and participants were asked to commit to daily practice between 40 and 60 min. The classes were modified from the original curriculum to remove discussions of depression relapse prevention and include a loving-kindness meditation in the final class, but no tinnitus-specific exercises or discussions were introduced.

### Behavioral Measures

#### Audiology

Participants received a complete audiological evaluation during an initial screening phase, as well as pre-intervention (week 0), post-intervention (week 8) and at an 8-week follow-up session. The full audiological screening and follow-up audiology assessments were reported in Husain et al. (2019).

The initial audiological screening included pure-tone audiometry (0.25–16 kHz) (Equinox 2.0 PC-based clinical audiometer) and is presented in Table 1 with the sample demographics.

#### Questionnaires

Three measures of tinnitus-related handicap were given at each session including the THI, TFI and Tinnitus Primary Function Questionnaire (TPFQ: Tyler et al., 2014). TFI, which has been shown to be sensitive to changes due to previous interventions (Henry et al., 2016), was chosen to be analyzed as a covariate of interest with functional MRI changes.

In addition to the tinnitus measures, the participants were given the MMSE, BDI, BAI, Big Five Personality Test (John et al., 1991), and Five Facet Mindfulness Questionnaire (FFMQ: Baer et al., 2008). TFI, BDI, and BAI were given at each of three MRI sessions. The behavioral data for this study was previously analyzed and reported in Husain et al. (2019) and provided evidence for significant improvement in self-reported tinnitus severity. The focus of this study was to examine the change in functional MRI measures across the MBCT intervention and to analyze the relationship between brain function and tinnitus severity as the variables change over the course of the study. Since our sample was so limited in this pilot study, we chose to focus on TFI as the primary measure of tinnitus severity.

### fMRI Scans

Two fMRI scans were used to acquire measures of brain function at pre-intervention, post-intervention, and follow-up sessions. First, a resting state scan was collected over approximately 10 min. During the resting state scans, participants were instructed to lay still and fixate on a cross for the duration of the scan. This task was used to analyze resting-state functional connectivity, and the change in connectivity across sessions.

The second scan was an affective sound categorization task, where participants were asked to rate sounds presented to them as pleasant, unpleasant, or neutral. Sounds were used from the International Affective Digital Sounds Database (IADS; Bradley and Lang, 2007), which include normative scores of valences (1 very unpleasant – 9 very pleasant) and arousals (1 low arousing – 9 high arousing). Only the normative valence score was used to categorize sounds into emotionally salient, including a total of 46 pleasant sounds (valence: 6.89 ± 0.25; arousal: 5.99 ± 0.27) and 46 unpleasant sounds (valence: 2.66 ± 0.38; arousal 6.95 ± 0.31), and emotionally neutral, including 46 neutral sounds (valence: 4.96 ± 0.36; arousal 4.92 ± 0.29), for analysis. At each session, a different combination of 46 sounds was played, matched for valence across sessions. Each sound in a session was played twice, totaling 92 sounds played per session. The full list and order of the sounds presented in each session is included in Supplementary Table 2. Sounds were delivered using Presentation software^1^ on a Windows 7 machine, with an Avotec silent scan 3300 sound system^2^ through sound dampening headphones.

### MRI/fMRI Acquisition

MRI data were collected on a 3T Siemens MAGNETOM Prisma MRI scanner with a 20-channel Siemens head coil.

An EPI sequence was collected during the resting state scan with parameters: TR = 2000 ms, TE = 25 ms, flip angle = 90°, 38 slices, 2.5 mm × 2.5 mm × 3.0 mm, FOV = 230 mm × 230 mm, matrix size = 92 × 92, with 304 volumes.

A second EPI sequence was collected during the auditory emotion task using a sparse sampling design in order to allow enough time for the auditory stimuli to evoke a BOLD response that does not overlap with the auditory response to the scanner gradients. The sequence had parameters: TR = 9000 ms (7000 ms delay), TE = 25 ms, flip angle = 90°, 38 slices, voxel size = 2.5 mm × 2.5 mm × 3.0 mm, FOV = 230 × 230, matrix size = 92 × 92, 92 volumes.

In addition, a low-resolution T2-weighted structural image (TR = 3400 ms, TE = 65.0 ms, flip angle = 120°, 38 slices, voxel size = 1.2 mm × 1.2 mm × 3.0 mm, FOV = 230 × 230, matrix size = 192 × 192) and a high-resolution T1-weighted MPRAGE (TR = 2300 ms, TE = 2.32 ms, flip angle = 8°, 192 slices, voxel size = 0.9 mm × 0.9 mm × 0.9 mm, FOV = 230 × 230, matrix size = 256 × 256) were collected and used for co-registration.

### fMRI Analysis

Two types of functional imaging analyses were conducted. First, results from the emotion task were analyzed. In the emotion task, the whole brain response to emotionally salient contrasted against emotionally neutral stimuli was analyzed for change throughout the intervention. Then, results from resting state data across the three intervention imaging sessions were analyzed. For the resting state data, the connectivity between the brain and several seed regions was analyzed for change throughout the intervention was analyzed, as well as how patterns of connectivity predicted TFI. In addition, how some graph measures of connectivity including modularity and efficiency changed across the sessions in the intervention was analyzed.

#### Affective Sound Categorization Task

Functional MRI data collected during the affective sound categorization task was analyzed using SPM12 software (Statistical Parametric Mapping, Welcome Trust Center for Neuroimaging^3^). Images were preprocessed in a five-step procedure including slice timing correction, realignment, coregistration, normalization, and smoothing (Gaussian kernel of 8 mm × 8 mm × 8 mm).

In the first level analysis, emotionally salient vs. neutral contrast images were generated for each subject for each session. Second level analysis analyzed sample-wide within session salient vs. neutral contrasts and contrasts between each of the session pairs using a paired *t*-tests. Cluster-level significance in the salient vs. neutral contrasts was assessed using a cluster-defining height threshold of *p* < 1e-5 and family-wise error (FWE)-corrected (*p* < 0.05) cluster extent defined by the SPM12 software through random field theory. Results found by reducing the height threshold to a more liberal *p* < 1e-4 are additionally cautiously presented.

#### Resting State Functional Connectivity

Functional MRI data collected during the resting state task was also analyzed using SPM12 software. Images were preprocessed using the same five-step procedure described above, but with some additional denoising steps implemented with the Functional Connectivity Toolbox (Conn)^4^ (RRID:SCR_009550) for MATLAB. These denoising steps included filtering the fMRI data with a 0.008–0.08 kHz bandpass filter and regressing out the signal from the white matter and cerebrospinal fluid segmentations from SPM12.

After denoising, seed-to-voxel connectivity analysis was conducted across several seeds of interest. Certain seed locations representing the default mode network (DMN) and the dorsal attention network (DAN) were the same as those used in earlier studies in our lab for comparison (Schmidt et al., 2013). In similar fashion to the study conducted by Schmidt et al. (2013) the DAN was split into two separate networks. The DMN used 8-mm spherical seeds corresponding to the medial prefrontal cortex and posterior cingulate cortex. The DAN_1 network used 8-mm spherical seeds corresponding to the left and right posterior intraparietal sulci. The DAN_2 network used 8-mm spherical seeds corresponding to the left and right frontal eye fields. Additionally, a seed representing the bilateral amygdalae was used given the evidence for its role in tinnitus (Carpenter-Thompson et al., 2015; Zimmerman et al., 2018). This seed was generated using the left and right amygdala parcellations in the atlas provided within the Conn toolbox, from the Harvard-Oxford subcortical atlas. For each of these regions, the seeds were combined prior to the connectivity analysis.

In addition to these networks, some network connectivity was investigated in an attempt to replicate and expand on results reported by Roland et al. (2015), which investigated changes in functional connectivity in the fronto-parietal (FPN) and cingulo-opercular networks (CON). As analyzed by Roland et al. (2015), seeds for these networks were separated by hemisphere. Seeds representing the left and right FPN in the left and right prefrontal cortex and representing left and right CON in the left and right anterior insulae were taken from the canonical network atlas provided in the Conn toolbox. The seeds and their coordinates in MNI space for each connectivity analysis are provided in Table 2.

**TABLE 2:** Seeds for seed-voxel connectivity analysis.

**Seed**	**Region**	**MNI Coordinate**
DMN	Medial prefrontal cortex	8, 59, 19
	Posterior cingulate cortex	−2, −50, 25
DAN_1	Left posterior intraparietal sulcus	−23, −70, 46
	Right posterior intraparietal sulcus	26, −62, 53
DAN_2	Left frontal eye field	−25, −11, 54
	Right frontal eye field	27, −11, 54
AMGY	Left amygdala	Harvard-Oxford atlas parcellation
	Right amygdala	Harvard-Oxford atlas parcellation
L_FPN	Left prefrontal cortex	−43, 33, 28
R_FPN	Right prefrontal cortex	41, 38, 30
L_CON	Left anterior insula	−44, 13, 1
R_CON	Right anterior insula	47, 14, 0

Cluster-level significance in seed-to-voxel connectivity was assessed using a height threshold of *p* < 1e-5, and FWE-corrected cluster extent defined by the SPM12 software through random field theory. Results found by reducing the height threshold to a more liberal *p* < 1e-4 are additionally cautiously presented.

#### Relationship With TFI

Generalized estimating equations (GEE) were used to estimate the extent to which TFI predicted brain function across the intervention. For both the affective sound categorization task and the resting state functional connectivity, TFI was used as a predictor at each voxel to predict the results from the first-level, within-subject contrasts in a multi-scale adaptive generalized estimating equations (MAGEE) model developed by Li et al. (2013).

MAGEE integrates the GEE approach with adaptive smoothing methods to make robust estimates of betas for each voxel. GEE models account for the within-subject correlation among the longitudinal measures through the specification of a correlation structure to estimate parameters, avoiding violations of the sphericity assumptions in analysis of variance (ANOVA) and violations of the assumption of equal observations per time point in multivariate ANOVA. GEE can thus use incomplete data under less stringent assumptions while accommodating time-varying covariates without assumptions about the structure of the covariance, improving the power of the analysis for longitudinal data (Diggle et al., 2002).

The multi-scale adaptive smoothing smooths over the beta estimates across a user set number of iterations (in this case 5), allowing for a balance between cluster size and peak intensities. The beta for the TFI predictor was tested against the null hypothesis that beta = 0. The MAGEE analysis outputs a map of the Wald statistic for that parameter estimate, which is compared against a chi-squared distribution with 1-degree of freedom. Cluster-level analysis was completed using the peak_nii toolbox^5^. Because of the increased power afforded by GEE, cluster-level significance was defined using a height threshold of *p* < 1e-5 and a cluster extent of 100. This height threshold and cluster extent were chosen cautiously to in order to restrict our analysis to relatively large and extended effects. However, results found by reducing the height threshold to a more liberal *p* < 1e-4 are additionally cautiously discussed. All statistical maps are presented after surface-mapping volumetric results using Connectome Workbench^6^ (Marcus et al., 2011).

#### Graph Analysis

To obtain a whole-brain functional network from the resting state fMRI data, first, the mean region of interest (ROI) time series were obtained by averaging the fMRI time series over all voxels within an ROI. For this purpose, 90 cortical and subcortical ROIs (excluding the ones from the cerebellum and vermis regions) defined in the Automated Anatomical Labeling (AAL) atlas (Tzourio-Mazoyer et al., 2002) were chosen. Then a 90 × 90 pairwise correlation matrix was obtained using the time series from these 90 regions. The pairwise correlation matrix was thresholded at various target connection densities to convert it to an undirected and unweighted (binary) connection matrix. In each case, correlations whose absolute values were higher than the threshold were converted to 1 s and the remaining correlations were converted to 0 s. At low graph connection densities, only the strongest correlations are present in the graph as connections, and as the graph density increases, weaker correlations also get added to the graph. As is standard in the literature, there is no *a priori* preference for a target connection density and so graph properties at a number of such connection densities were investigated.

##### Static network analysis

First, a single static network for each subject was created using the resting state time series data from the whole imaging session. With these static networks, the subjects’ brain networks at pre, post, and follow-up sessions were compared both at the whole network level as well as at ROI level. Since data at all three sessions were available for only eight subjects, the remaining subjects’ data were excluded from this analysis. The subjects’ brain networks at pre, post and follow-up sessions were analyzed in terms of three commonly employed global graph summary measures, modularity, global network efficiency, and global clustering coefficient, and also tested for association of changes in these measures with changes in TFI scores between consecutive sessions.

Modularity is a quality function that measures how much the networks are modular, i.e., segregated into communities or modules as opposed to what one might expect from a random network with similar node degree patterns. The Newman–Girvan modularity is computed using the Louvain algorithm for community detection (Blondel et al., 2008). The global network efficiency is defined as the average of the inverse shortest paths between pairs of vertices in the entire network (Rubinov and Sporns, 2010). The smaller the shortest paths are between pairs of vertices in the network, the higher the global efficiency. Intuitively, shorter paths between pairs of vertices allow information to pass throughout the network more efficiently. More locally, the nodal network efficiency of node *i* is defined as the average of the inverse shortest paths of all nodes in the network from the node *i*. Finally, the local clustering coefficient for a vertex measures the ratio of pairs of neighbors of the vertex *i* that are themselves connected (thus forming a closed triplet or triangle), to the number of such possible connections. The global clustering coefficient is defined as the average of the local clustering coefficient across the vertices of the network. The clustering coefficient is a measure of network segregation (Rubinov and Sporns, 2010), and a large clustering coefficient indicates that an otherwise sparse network is locally clustered and dense. For the purpose of statistical testing, due to the small sample size, non-parametric tests were employed along with parametric tests. All graph analyses were carried out in the statistical analysis software R.

Beyond global summary measures, the subjects’ brain network community structures between pre and post sessions were also compared by fitting the Random Effects Stochastic Block Model (Paul and Chen, 2018) with the Co-regularized Orthogonal Symmetric Non-negative Matrix Tri-Factorization (Co-OSNTF) method (Paul and Chen, 2018). The number of communities to be used with the Co-OSNTF method was obtained by taking the median of the number of communities detected in the subject networks separately using the Louvain method for community detection. The Co-OSNTF method was used to statistically test for differences in the global community structure as well as identify ROIs that have significantly different community placements between the two time sessions. Further, subjects’ brain networks were compared at each ROI in terms of nodal efficiency and local clustering coefficient in an effort to identify the ROIs which exhibit differential properties.

##### Dynamic network analysis

Time varying networks from the ROI time series data for each subject at each session were constructed using a sliding window approach (Hutchison et al., 2013). A window size of 20 TRs (40 s) was moved by 1 TR to obtain 280 time windows. For each window, a correlation matrix is estimated from the data within that window. The absolute correlation was thresholded to obtain a sparse connection matrix with connection density of 0.10. The subjects’ networks were then compared at pre and post intervention in terms of dynamic fluctuations of network efficiency, modularity and clustering coefficient.

## Results

Paired *t*-tests showed that TFI scores were significantly lower at the post-intervention compared to pre-intervention sessions [*t*(9) = 4.97, *p* < 0.001] and significantly lower at the follow-up session compared to the pre-intervention session [*t*(7) = 4.67, *p* = 0.002]. However, there was no significant difference between the post-intervention and follow-up sessions [*t*(7) = 0.45, *p* = 0.66]. Table 3 shows the mean and standard deviations of TFI scores at each session as well as the hours of logged weekly MBCT practice for each session.

**TABLE 3:** Tinnitus Function Index and mindfulness practice.

**TFI**	**Mean**	***SD***
Pre-intervention	50.92	15.57
Post-intervention	35.40	15.80
Follow-up	35.38	21.07

**Minutes of practice**	**Mean**	***SD***

During MBCT	960.60	510.20
After MBCT	605.50	210.11

There was a large variance in hours of practice of the 8-week period of intervention and the 8-week period following the treatment. Despite a numerical decline in mean practice hours, there was not a statistically significant difference between the amount of mindfulness practice logged during the MBCT and during the 8-week period after the MBCT ended [*t*(7) = 2.05, *p* = 0.08].

### Affective Sound Categorization Task

There was little evidence for changes in functional activation during the affective sound categorization task across the three sessions of the study (Supplementary Figure 1). There were no statistically significant changes between sessions for the salient vs. neutral contrasts.

Additionally, there was no evidence for the activation differences between salient and neutral conditions predicting TFI in the participants (Supplementary Figure 2).

However, results from the analysis at more liberal thresholds were also reported as possible points of follow-up for future studies, although these results should be interpreted with caution. Peak MNI coordinates and the direction of the relationship for each test are included in Table 4. There was only some suggestive evidence, after reducing the statistical threshold, for a change in functional activation to the salient vs. neutral contrast between the post-intervention and follow-up sessions, where a decreased activation in clusters peaking in the left inferior frontal gyrus and the right insula were observed. Patterns of functional activation to the affective sound categorization task were not significantly predicted by TFI, even after reducing the statistical threshold.

**TABLE 4:** Results from the analysis of the affective sound categorization fMRI.

**Region**	**Peak MNI coordinates**	**Beta direction**
*Post vs. Pre*		
None		
*Follow-up vs. Pre*		
None		
*Follow-up vs. Post*		
L. inferior frontal gyrus, pars opercularis^*^	−52, 16, 18	–
L. insula^*^	−42, 14, 6	–
*Prediction of TFI*		
None		

^*^p < 1e-4 height threshold.

### Resting State Functional Connectivity

For the resting state analysis, we assessed how the seed-to-voxel whole brain connectivity changed across the MBCT intervention. We analyzed pre-determined seeds, meant to reflect the connectivity with the DMN, DAN_1 and DAN_2, AMYG, left and right FPN, and left and right CON. Significant results are reported in the main text, but for all of the networks, the full connectivity maps for each session, the full statistical maps of all non-significant between session changes, and the non-significant statistical maps of the connectivity associated with TFI are all presented in supplement (Supplementary Figures 3–14).

#### Default Mode Network

**FIGURE 1:**
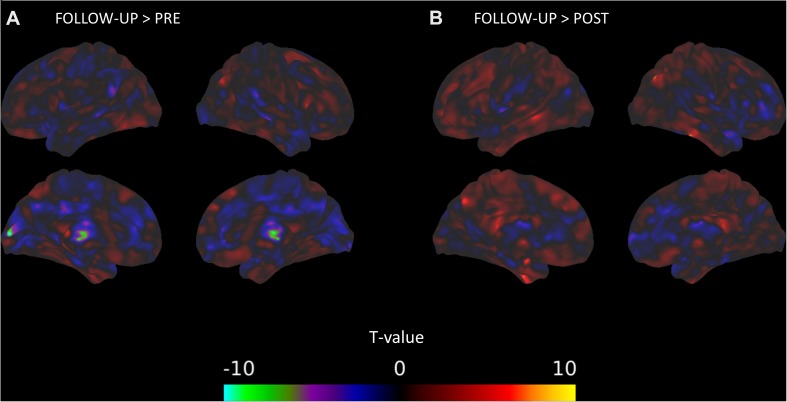
DMN connectivity changes between time points of the mindfulness intervention. **(A)** Connectivity between the DMN and clusters in the right thalamus and left calcarine sulcus significantly decreases from the pre-intervention session to the follow-up session. **(B)** Connectivity between the DMN and a cluster in the right angular gyrus significantly increases from the post-intervention session to the follow-up session.

The between-session comparisons revealed a pattern of decreased connectivity between the DMN and the right thalamus and occipital regions from pre-intervention to the follow-up session (Figure 1A). In contrast, there were connectivity increases between the DMN and clusters with peaks in the right angular gyrus and left superior temporal gyrus from the post-intervention session to the follow-up session (Figure 1B). There was some evidence that connectivity between the right middle frontal gyrus and the DMN and between the right putamen and the DMN was positively related with TFI, but there was no indication that connectivity with the DMN and these areas changed across the intervention (Table 5).

**TABLE 5:** Results from the analysis of the DMN.

**Region**	**Peak MNI coordinates**	**Beta direction**
*Post vs. Pre*		
L. middle temporal gyrus^*^	−60, −42, −4	–
*Follow-up vs. Pre*		
L. calcarine sulcus^∗∗^	2, −92, 6	–
R. thalamus^∗∗^	2, −14, 8	–
*Follow-up vs. Post*		
R. angular gyrus^∗∗^	40, −70, 38	+
L. middle temporal gyrus^*^	−70, −36, 4	+
*Prediction of TFI*		
R. middle frontal gyrus^*^	36, 6, 62	+
R. putamen^*^	32, −4, −2	+

^∗∗^p < 1e-5 height threshold, ^*^p < 1e-4 height threshold.

#### Amygdala

**FIGURE 2:**
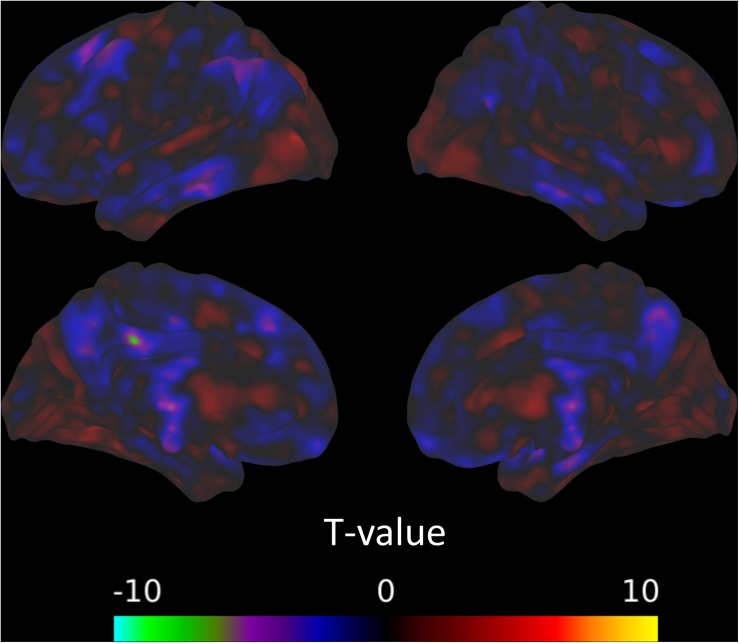
Connectivity with the amygdala from the pre-intervention session to the post-intervention session decreases in a cluster overlapping the left inferior parietal lobule.

**FIGURE 3:**
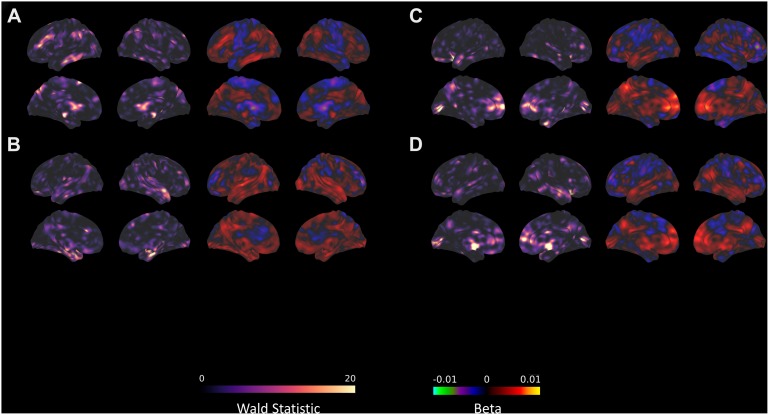
Seed-voxel connectivity that significantly predicts TFI with the **(A)** AMYG, **(B)** DAN_1, **(C)** left FPN, and **(D)** right FPN. For each section, the left panel shows the Wald statistic, while the right panel indicates the beta value and the direction of the relationship.

There was a significant decrease in connectivity between a cluster overlapping the left inferior parietal lobule and the amygdala from the pre-intervention session to the post-intervention session (Figure 2). There was also evidence for the connectivity between the amygdala and multiple areas in the brain, mostly in frontal and parietal areas, relating positively to TFI (Figure 3A). The combined observations of the amygdala connectivity with parietal areas is intriguing. On one hand, the connectivity between these two regions significantly and positively predicts tinnitus severity. On the other hand, the connectivity between these regions significantly decreases over the intervention, suggesting that the decreasing connectivity may reflect some of the benefits of the MBCT. Table 6 completely summarizes the statistical tests for changes in each session and for connectivity associated with TFI.

**TABLE 6:** Results from the analysis of the AMYG.

**Region**	**Peak MNI coordinates**	**Beta direction**
*Post vs. Pre*		
Left inferior parietal lobule^∗∗^	−42, −58, 54	–
*Follow-up vs. Pre*		
None		
*Follow-up vs. Post*		
None		
*Prediction of TFI*		
L. precuneus^∗∗^	−4, −78, 47	+
L. inferior frontal gyrus – pars triangularis^∗∗^	−48, 40, 12	+
L. inferior frontal gyrus – pars triangularis^∗∗^	−48, 36, 20	+
L. inferior frontal gyrus – pars triangularis^*^	−32, 36, 12	+
Undefined^*^	−26, −48, 14	−
Undefined ^*^	−18, −42, 16	−
L. middle frontal gyrus^*^	−50, 22, 36	+
L. precentral gyrus^*^	−50, 10, 42	+
L. precentral gyrus^*^	−44, 12, 34	+
L. middle frontal gyrus^*^	−38, 20, 54	+
L. middle frontal gyrus^*^	−36, 30, 48	+
Undefined^*^	6, 0, 14	–
Undefined^*^	0, 6, 4	–
L. superior parietal lobule^*^	−32, −64, 46	+

^∗∗^p < 1e-5 height threshold, ^*^p < 1e-4 height threshold.

#### Dorsal Attention Network

There were no changes in connectivity with either of our DAN divisions in any of the pairwise session comparisons at the established threshold for significance. Even after reducing the voxel height threshold to be more liberal, no clusters emerged. Despite the lack of change in connectivity over the intervention, there were several clusters where the connectivity with DAN_1 significantly correlated with TFI (Figure 3B), primarily in the medial temporal lobe and cerebellum (Table 7).

**TABLE 7:** Results from the analysis of the DAN.

**Region**	**Peak MNI coordinates**	**Beta direction**
**DAN_1**		
*Post vs. Pre*		
None		
*Follow-up vs. Pre*		
None		
*Follow-up vs. Post*		
None		
*Prediction of TFI*		
Undefined^∗∗^	16, −14, −28	+
R. parahippocampal gyrus^∗∗^	22, −10, −24	+
R. parahippocampal gyrus^∗∗^	26, −4, −32	+
L. lobule IV/V of cerebellar hemisphere^∗∗^	−30, −36, −30	+
Undefined^∗∗^	−14, −32, −34	+
L. parahippocampal gyrus^∗∗^	−24, −6, −28	+
L. hippocampus^∗∗^	−22, −20, −16	+
Undefined^*^	12, −34, −30	+
L. parahippocampal gyrus^*^	−20, 8, −28	+
L. inferior temporal gyrus^*^	−34, 8, −38	+
R. superior temporal gyrus^*^	46, −28, 12	+
Undefined^*^	34, −32, 12	+
Undefined^*^	22, −38, 0	+
R. hippocampus^*^	20, −30, −6	+
**DAN_2**		
*Post vs. Pre*		
None		
*Follow-up vs. Pre*		
None		
*Follow-up vs. Post*		
None		
*Prediction of TFI*		
L. superior occipital gyrus^*^	−14, −74, 22	+
R. inferior frontal gyrus, pars orbitalis^*^	41, 29, −5	+

^∗∗^p < 1e-5 height threshold, ^*^p < 1e-4 height threshold.

In contrast with DAN_1, no connectivity with DAN_2 significantly predicted TFI. However, by reducing the height threshold, two suggestive clusters appeared within inferior frontal and superior occipital regions (Table 7).

#### Fronto-Parietal Network

There were no changes in connectivity with the left FPN in any of the pairwise session comparisons at the established threshold for significance, although there was some evidence for decreased connectivity between the right FPN and left caudate nucleus from pre- to post-intervention when reducing height threshold. Despite the lack of change in connectivity over the intervention, there were several clusters where the connectivity with the FPN significantly correlated with TFI (Table 8). These included lobule IX of the cerebellar hemisphere, the bilateral insulae, right hippocampus, right middle temporal gyrus, and the right inferior frontal gyrus (Figures 3C,D). In contrast, connectivity between the left FPN and the left calcarine sulcus was significantly negatively correlated with TFI (Figure 3C).

**TABLE 8:** Results from the analysis of the left and right FPN.

**Region**	**Peak MNI Coordinates**	**Beta direction**
**Left FPN**		
*Post vs. Pre*		
None		
*Follow-up vs. Pre*		
None		
*Follow-up vs. Post*		
None		
*Prediction of TFI*		
L. calcarine sulcus^∗∗^	0, −84, 0	−
Undefined^∗∗^	−14, −86, 2	+
R. lobule IX of cerebellar hemisphere^∗∗^	12, −50, −46	+
L. insula^∗∗^	−29, 19, −17	+
R. hippocampus^∗∗^	29, −35, 0	+
R. lobule VIII of cerebellar hemisphere^*^	18, −64, −38	+
L. medial frontal gyrus^*^	−6, 62, 4	+
R. anterior cingulate gyrus^*^	8, 46, 8	+
L. anterior cingulate gyrus^*^	−8, 36, 2	+
R. anterior cingulate gyrus^*^	2, 37, −1	+
L. medial frontal gyrus^*^	−8, 48, 6	+
**Right FPN**		
*Post vs. Pre*		
L. caudate nucleus^*^	−10, 12, −4	–
*Follow-up vs. Pre*		
None		
*Follow-up vs. Post*		
None		
*Prediction of TFI*		
Undefined^∗∗^	0, −2, −10	+
Undefined^∗∗^	15, −25, −37	-
R. middle temporal gyrus^∗∗^	56, −6, −18	+
R. middle temporal gyrus^∗∗^	52, −18, −12	+
R. insula^∗∗^	33, 16, −12	+
R. inferior frontal gyrus – pars orbitalis^∗∗^	34, 22, −22	+
Undefined^*^	26, −16, −6	+
R. lobule IX of cerebellar hemisphere^*^	10, −52, −40	+
L. lobule IX of cerebellar hemisphere^*^	−6, −52, −40	+
R. calcarine sulcus^*^	28, −64, 12	–
R. calcarine sulcus^*^	14, −80, 8	+
L. calcarine sulcus^*^	−8, −96, 0	+
L. calcarine sulcus^*^	−2, −88, 0	+
R. superior temporal pole^*^	46, 18, −22	+
R. medial frontal gyrus^*^	6, 56, 8	+
R. anterior cingulate gyrus^*^	6, 42, 4	+

^∗∗^p < 1e-5 height threshold, ^*^p < 1e-4 height threshold.

#### Cingulo-Opercular Network

**FIGURE 4:**
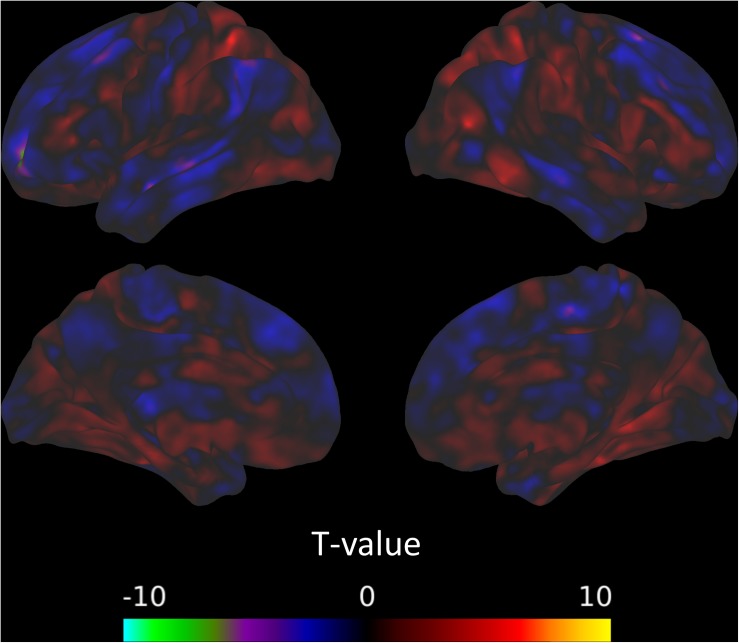
Connectivity between the left CON and the left superior frontal gyrus decreases from the post-intervention to the follow-up session.

The between-session comparisons revealed a pattern of decreased connectivity between the left CON and the left superior frontal gyrus from the post-intervention to the follow-up session (Figure 4). However, there was no indication that connectivity with the CON changed across the intervention (from the pre-intervention to the post-intervention sessions) or was predicted by TFI (Table 9).

**TABLE 9:** Results from the analysis of the left and right CON.

**Region**	**Peak MNI coordinates**	**Beta direction**
**Left CON**		
*Post vs. Pre*		
None		
*Follow-up vs. Pre*		
None		
*Follow-up vs. Post*		
L. superior frontal gyrus^∗∗^	−32, 62, −2	−
R. lobule VI of cerebellar hemisphere^*^	34, −38, −38	+
*Prediction of TFI*		
None		
**Right CON**		
*Post vs. Pre*		
None		
*Follow-up vs. Pre*		
None		
*Follow-up vs. Post*		
R. calcarine sulcus^*^	18, −52, 8	+
*Prediction of TFI*		
None		

^∗∗^p < 1e-5 height threshold, ^*^p < 1e-4 height threshold.

#### Global Graph Properties

**FIGURE 5:**
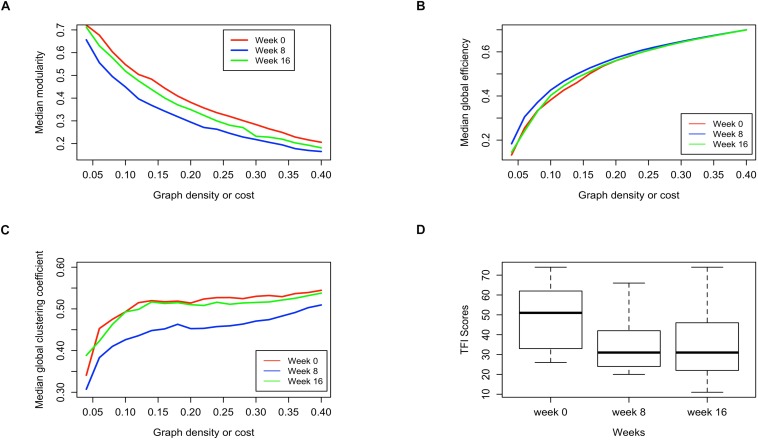
Comparison of pre-intervention (week 0), post intervention (week 8) and follow up (week 16) session brain networks in terms of **(A)** Median modularity, **(B)** Median global efficiency, and **(C)** Median global clustering coefficient as a function of increasing network density, along with the **(D)** distribution of the TFI scores measured for subjects at those sessions.

Figures 5A–C present the median modularity, global efficiency, and global clustering coefficient respectively, at pre-intervention, post-intervention and follow-up (weeks 0, 8, and 16), at various graph density thresholds or costs. From Figure 5A it appears that the modularity of the brain networks decreases post-intervention as compared to pre-intervention, but returns closer to the previous level at the follow-up session. There are similar trends in global efficiency and global clustering coefficient. While global efficiency increases at the post-intervention session compared to pre-intervention, the measure returns close to the previous level at the follow-up session (Figure 5B). This phenomenon is even more pronounced with global clustering coefficient, which is substantially lower at the post-intervention compared to pre-intervention, but almost entirely returns to the previous levels at the follow-up session (Figure 5C). These observations largely hold across different brain network density or cost. Hence, at the post-intervention session, the subject networks tend to be less functionally segregated (Rubinov and Sporns, 2010) as evident with lower modularity and clustering coefficient, and more functionally integrated (Rubinov and Sporns, 2010) as evident by higher global efficiency. However, both these changes to the brain networks tend to return to pre-intervention levels by the follow-up session.

Remarkably, the distribution of TFI scores show a similar phenomenon, where the scores drop substantially at the post-intervention compared to pre-intervention, but remain stationary at the post-intervention level when measured again during the follow-up session (Figure 5D). The distribution of TFI scores was calculated over all subjects for whom data were available at the respective stage of data collection. Based on these observations we hypothesized that the changes in TFI scores might be related to the changes in the brain network global summary measures.

As reported in Table 10, the difference in TFI scores was found to be positively correlated with changes in both the measures of functional segregation (modularity and clustering coefficient) with the correlation being statistically significant [0.05 family-wise error rate (FWER), corrected for multiple comparisons] at lower graph densities of 0.05 and 0.10 (significant in all but one graph density without correction for multiple comparisons). Notably, the correlations between the change in functional segregation measures and change in TFI scores are stronger at lower graph densities. The association between the difference in TFI scores and global network efficiency was not significant at any graph density. Additional analysis of the global graph properties are provided in supplement, including normalizing measures of inter-session changes (Supplementary Table 3), analyzing a regression model of changes in TFI using global graph measures as predictors (Supplementary Table 4) and analyzing multiple density thresholds (Supplementary Table 5 and Supplementary Figure 15). Furthermore, dynamic changes in efficiency, modularity and clustering are provided (Supplementary Figure 17), as well as their distribution smoothed by a kernel density estimator (Supplementary Figure 18).

**TABLE 10:** Correlation of TFI score differences with the differences in modularity and clustering coefficient.

**Graph density**	**Modularity (*p*-value)**	**Clustering coefficient (*p*-value)**
0.05	0.6851 (0.0034^∗∗^)	0.6597 (0.0054^∗∗^)
0.10	0.6377 (0.0078^∗∗^)	0.6688 (0.0046^∗∗^)
0.15	0.4915 (0.0531)	0.4518 (0.0789)
0.20	0.5316 (0.0340^*^)	0.5355 (0.0325^*^)
0.25	0.5271 (0.0358^*^)	0.5073 (0.0448^*^)

^∗∗^p < 0.01 levels (p < 0.05, FWER level), ^*^p < 0.05.

#### Local Graph Properties

The subjects’ networks at pre- and post-intervention were compared in terms of nodal efficiency and local clustering coefficient at each of the 90 ROIs using two-sample *t*-tests. At a connection density of 0.10, two ROIs, the right middle frontal gyrus, orbital part (Frontal_Mid_Orb_R), and the right angular gyrus (Angular_R) were found to exhibit significantly different nodal efficiency with a false discovery rate (FDR) correction of 5% (the ROI Frontal_Mid_Orb_R was also significant at 5% FWER). One ROI, the right calcarine fissure (Calcarine_R), was found to exhibit a significantly different local clustering coefficient with an FDR correction of 5% (also significant at 5% FWER).

At a connection density of 0.20, no ROIs were found to exhibit any significantly different nodal efficiency with an FDR correction of 5%. However, two ROIs, the right medial superior frontal gyrus (Frontal_Sup_Medial_R), and the right middle occipital gyrus (Occipital_Mid_R), exhibited significantly different local clustering coefficients with an FDR correction of 5%. At pre-intervention, some ROIs were found to exhibit very low nodal efficiency, indicating a disconnect of those ROIs with the rest of the network. However, those ROIs exhibited nodal efficiency at par with other ROIs post intervention.

Next, random effects stochastic block models (RESBM) were fitted using the Co-OSNTF method (Paul and Chen, 2018) to pre- and post-intervention subject networks at 0.20 connection densities. The median number of communities at pre-intervention brain networks was 5 and at post-intervention was 4.9 and hence 5 was selected to be the number of communities to be used with Co-OSNTF. The putative group module structures obtained from this method represent the module structures of all the 10 subjects at pre- and post-sessions and are presented in Supplementary Figure 16. The group module structures were found to be significantly different between pre- and post-intervention at the whole network level with *p* = 0.0086. However, no nodes were found to have significantly altered module membership at 5% FDR.

RESBM was also fitted to the pre and post-networks at 0.10 connection density with the number of communities as 6. The group module structures were found to be significantly altered between pre- and post-intervention with *p* = 0.0033. In addition, two ROIs, the left middle occipital gyrus (Occipital_Mid_L) and right inferior occipital gyrus (Occipital_Inf_R), were found to exhibit significantly altered module memberships in post-intervention as compared to pre-intervention with a 5% FDR correction (both ROIs were also significant with 5% FWER correction).

## Discussion

In this study, our focus was on explaining declines in tinnitus severity due to MBCT using task-based and resting-state fMRI. For the task-based fMRI, when the participants were processing affective sounds, we did not observe changes in the response of any of the brain regions at post-intervention or at follow-up. However, there were changes across the intervention in the resting state connectivity of a diverse set of regions, as well as evidence for certain patterns of functional connectivity relating to tinnitus handicap. Because we observed some overlap between changes across the intervention and a relationship to TFI, connectivity between the amygdala and parietal regions showed particular promise as an area involved in the improved tinnitus severity outcomes across the MBCT intervention. In contrast, connectivity with other ROIs that we investigated showed varying degrees of evidence for sensitivity to the intervention or as relating to TFI but lacked any overlap. This overlap is critical to interpreting the role of brain regions or connectivity benefiting from the intervention, since MBCT may induce functional brain changes that have no bearing on predicting tinnitus severity, and similarly, there may be functional patterns of brain activation that are predictive of tinnitus severity but are not influenced by the specific MBCT intervention.

Despite the sparse evidence for the resting connectivity with particular ROIs relating to the beneficial effects of the MBCT intervention (besides connectivity with the amygdala), global measures of connectivity from graph analysis did seem to have some correspondence to the decrease in tinnitus severity over the course of the intervention. Particularly, measures of functional segregation, modularity, and cross-correlation coefficient, which decreased over the MBCT intervention predicted the decreases in TFI.

### Changes in RSFC During MBCT

We found evidence for changes in seed-to-voxel connectivity across the MBCT intervention in the DMN, AMYG, and left CON, but did not see evidence in the DAN or left or right FPN.

Interestingly, in both the DMN and the CON, the strongest contrasts in connectivity appeared to involve the follow-up session. In the DMN, we observed significant decreases in connectivity with the left calcarine sulcus and right thalamus from pre-intervention to the follow-up session. We also observed increased connectivity with the right angular gyrus from the post-intervention session to the follow-up. There was weaker evidence for a decrease in connectivity with the left middle temporal gyrus over the intervention, but then an increase in connectivity in the same area from the post-intervention session to the follow-up, after the intervention had ended. It is notable that the most significant changes in connectivity with the DMN occurred in contrasts with the follow-up session, suggesting that the brain may continue adapting even after the intervention. Our data suggested that there is substantial variance in practice both during and after the treatment and that our participants as a group continued their practice even after the MBCT ended.

Our work replicates other studies of mindfulness that have found decreases in connectivity between the DMN and regions involved in visual processing (Kilpatrick et al., 2011). However, we did not see any evidence of increased connectivity across the intervention with the dorsolateral prefrontal cortex, which has been identified in other studies (Brewer et al., 2011; King et al., 2016a) and has been implicated in improvement with other conditions (e.g., post-traumatic stress disorder, King et al., 2016a). It should also be noted that although researchers have observed connectivity increases *within* the DMN (e.g., Jang et al., 2011), we chose to combine seeds in the posterior cingulate cortex and medial prefrontal cortex, which limits our ability to study any within network effects.

In the CON (sometimes called the salience network), we did not observe any changes across the intervention, but did observe a decrease in connectivity between the left CON and the left dorsolateral frontal gyrus between the post-intervention and follow-up sessions. The CON has been implicated in mindfulness-based treatments primarily in its increased connectivity with self-referential regions, such as the dorsomedial prefrontal cortex, and decreased connectivity with visual regions (Kilpatrick et al., 2011; Doll et al., 2015). Doll et al. (2015) found some trending evidence for increased connectivity between the salience network and what they refer to as the left central executive network, which included the left dorsolateral frontal gyrus, after 2 weeks of daily 20-min attention to breath training. Based on this, it seems more probable that the decrease in connectivity between post-intervention and follow-up sessions represents a return to baseline levels of connectivity after the intervention, rather than continued adaptation from the intervention. However, interpretation of any of the contrasts with the follow-up session, without significant effects at other contrasts, is very difficult, and highlights the importance of continuing this work with larger studies.

In the amygdala, we observed decreased connectivity with the left inferior parietal lobule from pre- to the post-intervention sessions. Changes in amygdala connectivity from mindfulness-based training treatments have been observed but seem to vary depending on the sample. For example, connectivity between the amygdala and anterior cingulate cortex decreased after a 3-day intensive mindfulness meditation intervention in a sample of stressed, unemployed adults (Taren et al., 2015). In a study on generalized anxiety disorder, Hölzel et al. (2013) found an increase in connectivity between the amygdala and prefrontal cortex over the course of an 8-week mindfulness-based stress reduction intervention, which correlated with the change in anxiety. King et al. (2016b) also found increased effective connectivity during an angry-faces viewing task between the left amygdala and medial frontal gyrus, dorsal anterior cingulate cortex, and lingual gyrus after a mindfulness-based exposure therapy on a sample of combat veterans with post-traumatic stress disorder. However, that study did not find significant correlations between changes in amygdala connectivity and symptom improvement.

Further research is required to determine whether connectivity changes with the amygdala are altered in a similar way across mindfulness interventions in accordance to a consistent framework or if instead changes in connectivity are highly dependent on the condition of the sample. For example, it may be possible to interpret connectivity changes across a mindfulness intervention in terms of increased cognitive control over emotional or default-mode processing, consistent across many forms of emotional dysregulation. Both research on post-traumatic stress disorder and existing research on tinnitus hypothesize that increased cognitive control over self-referential processing leads to improved outcomes (Malinowski, 2013; Husain, 2016; Schmidt et al., 2017).

Alternatively, there may be large condition specific effects. For example, in tinnitus, perhaps the emotional interpretation of primary sensory information is a critical aspect of the condition and is highlighted through changes in amygdala connectivity with the inferior parietal lobule. Conditions where the emotional interpretation of one’s social position is more critical may instead be highlighted through changes in amygdala connectivity with the cingulate cortex or prefrontal cortex.

A broader review of the neural bases of both MBCT and the related mindfulness-based stress reduction (Gotink et al., 2016) revealed increased activity and gray matter volume in the prefrontal cortex, the cingular cortex, the insula and the hippocampus in both individuals with stress or anxiety and healthy participants. In contrast, the amygdala showed decreased response. A more recent systematic review of fMRI studies of mindfulness-based interventions (Young et al., 2018) did not find compelling evidence of increased response in the prefrontal cortex; rather, the authors found significant increased insular cortex activity correlated with the longitudinal interventions. Again, the authors noted the variety of findings with respect to neural bases of mindfulness-based interventions dependent on the populations and tasks.

### Predicting Changes in TFI Using RSFC

One of the most striking results from this pilot study is the difference between functional connectivity that changes over a mindfulness intervention on one hand and functional connectivity that is predictive of tinnitus severity on the other. Connectivity between the FPN and broad regions of the brain predicted TFI, despite not showing any significant changes due to the intervention. Likewise, the DAN connectivity with a variety of regions, particularly limbic structures, predicted TFI, despite having no change due to MBCT. In contrast, connectivity to the DMN only weakly predicted TFI, and connectivity to the CON did not predict TFI.

We focused our analysis on resting state connectivity changes with regions that were implicated in both tinnitus and mindfulness-based treatments. Studies conducted on differences in functional connectivity in tinnitus compared to normal controls have identified changes in connectivity primarily in the DMN and networks involved in attention (Schmidt et al., 2013, 2017; Husain and Schmidt, 2014). While the results of this study largely replicate the importance of these networks in tinnitus, there were some key differences. Primarily, differences in DMN connectivity that predicted tinnitus have focused on the decreased connectivity between DMN and the precuneus (Schmidt et al., 2013, 2017), whereas this study only found weak evidence for increases in connectivity between the DMN and the right middle frontal gyrus and putamen as predictive of TFI. In addition, while the results here replicate past results (e.g., Schmidt et al., 2013) showing increased connectivity between the attention networks and limbic regions, our study additionally implicates increased connectivity between attention networks and the cerebellum, inferior frontal areas, and occipital areas in predicting TFI. Although connectivity with the DAN and FPN have similar patterns in predicting tinnitus, connectivity between the FPN and the right middle temporal gyrus additionally predicted TFI, which was an absent effect in the DAN. Connectivity with the amygdala also predicted TFI, but the effects were focused primarily to increased connectivity with frontal and parietal regions.

It should also be noted that the study here investigates how a measure of tinnitus severity, TFI, within a sample of individuals with tinnitus, predicts functional connectivity. This contrasts with most of the previous research, which focuses on differences between individuals with tinnitus vs. normal-hearing controls. It may be the case that changes in DMN connectivity strongly differentiate tinnitus patients from controls, but are far less important in predicting severity, whereas connectivity with regions involved in attention and emotion plays a far greater role in predicting severity.

### Functional Correlates of Successful MBCT

Thus far, we discussed the mostly disparate results between changes in connectivity across the intervention and connectivity that predicts tinnitus severity. However, for the purpose of understanding any benefits of the intervention in terms of functional connectivity, we would like to observe changes in connectivity that also predicted TFI. This key overlap was evident in connectivity between the amygdala and parietal regions and in some of the global graph theoretical metrics of connectivity.

The cluster of amygdala connectivity with the parietal areas that changed over the intervention peaked in the left inferior parietal lobule, whereas amygdala connectivity with parietal areas that predicted TFI peaked in the left precuneus and left superior parietal lobule. Future research will be required to determine if these patterns of connectivity represent the same mechanism of some broad connectivity between the amygdala and the parietal lobe or if they represent two distinct patterns of connectivity. Interpreting the connectivity with parietal areas is further complicated by the complex functions of the parietal lobe. In fact, even within the precuneus, functions, and connections may vary widely between dorsal and ventral regions (Cavanna and Trimble, 2006; Zhang and Li, 2012). Despite these difficulties, the observation is a promising lead for future research. A large region of decreased connectivity between the amygdala and the parietal lobe appears to overlap with regions where that change predicts tinnitus severity. This overlap represents the critical result from the functional connectivity analysis, since every other measure of functional connectivity that changes over the intervention did not show any relation to tinnitus severity. With such a small sample, this may simply be due to being underpowered, but it is intriguing to speculate about the specific role of amygdala-parietal connectivity in tinnitus. On the one hand, the connectivity could be interpreted in terms of emotional regulation through the control functions of the parietal lobe. However, previous research has shown *increased* connectivity between the amygdala and cognitive control networks in response to cognitive behavioral therapy (Shou et al., 2017) and in mindfulness practice (Doll et al., 2016). Therefore, we would predict that if it was an attentional effect, the change in connectivity and the relation to tinnitus severity would be in the opposite direction from what we observed. It is possible that the reduction in connectivity observed here instead reflects a reduced need to process self-referential information related to stress. Increased connectivity between amygdala and the precuneus has been seen in response to psychosocial stress (Veer et al., 2011). In addition, Aghajani et al. (2014) observed increased amygdala-precuneus connectivity related to neuroticism and speculated that aberrant self-referential information processing reflected in this connectivity difference, may increase the propensity toward negative emotions. Despite the decreasing overall connectivity between the amygdala and parietal areas, we did observe increasing local nodal efficiency over the intervention in frontal and parietal areas as well, which may represent increased efficiency of areas involved in cognitive control. More research is clearly needed to elucidate the meaning of these findings and more finely define the spatial effects.

Besides these ROI based results, there were also global measures of connectivity that predicted the change in TFI. Specifically, decreases in measures of functional segregation (modularity and clustering coefficient) predicted decreases in TFI. Previous works have suggested meditation or mindfulness training to be associated with a decreased functional segregation and increased function integration in the brain networks (Gard et al., 2014; van Lutterveld et al., 2017). Our observations in this study are in line with those findings. While we found mindfulness training to be associated with both decreased segregation and increased integration, only the changes in the measures of segregation were found to be correlated with changes in TFI scores. Again, it is important to point out that this change in functional segregation speaks to a functional change within a sample of individuals with tinnitus and without a control it is impossible to relate to a non-tinnitus group. The relationship is particularly intriguing, since research on intervention-related plasticity typically has found modularity to positively predict behavioral outcomes across a variety of interventions (see Gallen and D’Esposito, 2019 for a review). It is possible that in the context of tinnitus or other conditions of disordered emotional processing modularity represents a different mechanism or pattern of brain function. This intriguing possibility further emphasizes the need for future research with a non-tinnitus comparison group.

Prior to this study, only one study has investigated the effects of a mindfulness-based intervention on the resting-state connectivity in tinnitus patients (Roland et al., 2015). Unlike that study, we did not observe changes to the connections between the attention networks and other regions but did note differences between DMN seeds and other regions. Interestingly, we did not see evidence that either of these patterns of connectivity overlapped with regions predicting tinnitus severity over the course of the intervention. In their study, they did not explicitly examine changes in connectivity that were predicted by tinnitus severity. The difference in the intervention effects may have been due to the difference in protocols (MBCT vs. mindfulness-based stress reduction). However, it is also probable that the small sample sizes in both studies yielded less reliable results or were underpowered to observe the effects.

### Limitations and Future Directions

Despite the promise of the analysis of the study in informing future directions in research on tinnitus interventions, this pilot-study has some great limitations. First, this was an open-label study, with the participants aware of the treatment they were receiving and no control group. The lack of a control group is somewhat offset by the longitudinal nature of the study; with the baseline longitudinal data on their self-report tinnitus measures, each participant served as their own control in some regards. However, it is difficult to definitively attribute the effects to the MBCT, and the results may be due to other factors not considered here, including placebo effects.

A further major shortcoming of this study is the small sample size, further exacerbated by a high dropout rate. A small sample size is sometimes an unfortunate aspect of studies of medical conditions due to challenges in recruitment and retention. The current study did not include data from any participants that missed more than two classes, which may be too restrictive of a criterion for future research, especially if adherence to home practice is maintained. A second limitation of the small sample is the inability to model heterogenous aspects of the sample, including age. We have attempted to mitigate the small sample size by using statistical techniques that allow unequal sample sizes and focus on population-level effects (rather than within-subject effects) to improve the power of the fMRI analyses as much as possible. We also attempted to report the results in such a way that makes all of the results accessible rather than limiting the scope of the results to a single set criterion. However, due to the limited sample size, the results presented here should be taken as suggestive rather than definitive.

We plan to use findings from this pilot study to guide future research and theory building for tinnitus interventions. Mindfulness-based interventions appear to be a promising therapy for tinnitus and understanding why may open ways to enhance or substitute these effects. Randomized controlled clinical studies with larger sample sizes and control groups with and without tinnitus will help determine the answer to some of the questions that the research here brings up. First, increased power will help to make a stronger case for whether mindfulness-based therapies improve many conditions through a similar mechanism (e.g., improved attentional control over emotional processing) or whether there are unique, condition-specific effects that need to be considered while developing future interventions. Secondly, a non-tinnitus control group would be beneficial in further elucidating patterns of brain function that predict the condition of tinnitus itself and patterns of brain function that predict severity within the condition.

## Conclusion

In this feasibility study, we measured fMRI at three time points: before, after, and at follow-up of an 8-week long MBCT intervention. In an affective sound categorization task, no significant functional activation differences were observed between sessions, nor was the activation to emotionally salient compared to neutral stimuli significantly predictive of TFI. However, we found significant changes in resting state connectivity with the DMN, cingulo-opercular network and amygdala after the intervention, and patterns of resting state connectivity with amygdala, DAN and frontoparietal network significantly predicted tinnitus-related handicap. Critically, the overlap of decreased amygdala connectivity with parietal areas and the negative correlation between amygdala-parietal connectivity and TFI is suggestive of a brain imaging marker of successful treatment of tinnitus-distress. Additionally, we found an intriguing pattern of decreased functional segregation predictive of decreased TFI over the course of the MBCT intervention. Our findings support the use of brain imaging, specifically resting state functional connectivity analyses, to provide clinicians and researchers with supplementary information to better evaluate the efficacy of interventions and to design more focused experiments in future.

## Data Availability

The datasets generated for this study are available on request to the corresponding author.

## Ethics Statement

This study was carried out in accordance with the recommendations of the Institutional Review Board of the University of Illinois at Urbana–Champaign, with written informed consent from all subjects. All subjects gave written informed consent in accordance with the Declaration of Helsinki. The protocol was approved by the Institutional Review Board of the University of Illinois at Urbana–Champaign.

## Author Contributions

FH obtained funding, directed this study, designed the research, and edited the manuscript. BZ oversaw all analysis, analyzed both resting state and task-based fMRI data, and wrote the manuscript. MF helped design the study and recruited the participants. Both MF and SS collected the MRI data and ran the initial analysis. SP and YC helped conduct graph connectivity analysis, with SP taking the lead and contributing text and figures. KR helped design the study and screened the participants. YT conducted the audiological and behavioral assessments and related data analysis. All authors contributed to manuscript revision, and read and approved the submitted version.

## Conflict of Interest Statement

The authors declare that the research was conducted in the absence of any commercial or financial relationships that could be construed as a potential conflict of interest.

## Footnotes

1 http://www.neurobs.com

2 http://www.avotecinc.com

3 https://www.fil.ion.ucl.ac.uk/spm/software/spm12/

4 www.nitrc.org/projects/conn

5 http://www.nitrc.org/projects/peak_nii

6 https://www.humanconnectome.org
