# Supplementary material for: Functional Brain Changes During Mindfulness-Based Cognitive Therapy Associated With Tinnitus Severity
Source: Front Neurosci. 2019 Jul 24;13:747. doi: 10.3389/fnins.2019.00747 (PMC6667657; doi:10.3389/fnins.2019.00747)
Supplement: Supplementary file 1 [file Data_Sheet_1.docx]

Supplementary Material

## Global graph properties

A potential caveat for the observation reported in Table 10 could be the influence of differing baseline scores for the subjects. To account for the differences, the inter-session changes in TFI scores, efficiency, modularity, and clustering coefficient were normalized by the values of the quantities observed in the preceding session (i.e., for post-pre measures were normalized by pre and for follow-up-post measures were normalized by post) (Supplementary Table 3). While the direction of correlations between the normalized changes in the TFI scores with the normalized changes in efficiency, modularity, and clustering coefficients are the same as in the un-normalized case (namely the decrease in TFI scores are positively associated with decrease in modularity, clustering coefficient and increase in efficiency), the magnitudes are slightly diminished for all three graph summary measures. In addition, only the normalized change in clustering coefficient at graph densities 0.05 and 0.10 and normalized change in modularity at graph density 0.10 significantly correlates with normalized change in the TFI scores.

A regression model was analyzed with changes in TFI scores as the response variable and with modularity and clustering coefficients as explanatory variables. The regression was found to be significant at lower graph densities of 0.05 and 0.10 and explained 54% and 45% variation in the changes in TFI scores respectively (Supplementary Table 4).

Since there is a large difference between the brain networks in pre- and post-intervention sessions across all three measures, the statistical analysis to compare the subject networks was restricted to those two sessions, which allowed for the inclusion of two additional subjects to the analysis. Supplementary Figure 15 presents the median of the pairwise differences in the three global graph summary measures, modularity, efficiency and clustering coefficient between pre- and post-intervention for these 10 subjects. From this figure, it appears that at lower density graphs, when only the strongest connections survive, functional integration (efficiency) tended to be higher and functional segregation (modularity and clustering coefficient) tended to be lower in post-intervention compared to pre-intervention.

At each of the density thresholds, the difference in these three measures among the subjects in pre- and post-intervention was statistically tested through a paired two-sample t-test. In addition to the two-sample paired t-test, the nonparametric Wilcoxon rank sum test was used due to concerns about the validity of the asymptotic results under low sample size and possible non-normality of the distribution of the quantities of interest. The results from the tests at five graph connection densities are presented in Supplementary Table 5. For all the graph connection densities that the global network measures were investigated at, there was a significant increase in global network efficiency, and a significant decrease in graph modularity and global clustering coefficient post intervention (week 8) as compared to pre-intervention (week 0). This implies that post intervention the subjects’ brain networks tend to be less segregated and more integrated.

.

**1.2 Comparison in terms of dynamic network properties**

Dynamic changes in efficiency, modularity and clustering coefficient were contrasted over all the 280 network snapshots between pre- and post-intervention sessions for each of the 10 subjects in terms of the time courses (Supplementary Figure 17), as well as their distribution smoothed by a kernel density estimator (Supplementary Figure 18). It is apparent from Supplementary Figure 18a that for all but one subject, the dynamic network efficiencies are generally higher in post-intervention as opposed to pre-intervention. Assuming the network snapshots within a subject to be independent samples, a paired t-test confirmed higher means for post-intervention efficiencies in 7 of the 10 subjects at a 5% FWER. Interestingly, the distributions in post-intervention have less spread compared to that of pre-intervention indicating lower variance. Hence, it appears that after mindfulness training the subjects had lower variation in global brain network efficiency over the resting state fMRI time course. A formal F-test for equality of variances confirmed higher variance at pre-intervention compared to post-intervention in 8 of the 10 subjects correcting for multiple comparisons using a 5% FWE rate. This is possibly because the periods of unusually low efficiency observed in the pre-intervention dynamic networks is somewhat alleviated after intervention (Supplementary Figure 18a). The differences are not as pronounced in modularity, while clustering coefficient appears to be generally lower in post-intervention in most network snapshots (Supplementary Figure 18c), and the distribution of clustering coefficient post intervention is slightly left-shifted (Supplementary Figure 18c). Graphically, no substantial differences in variation of modularity and clustering coefficient over the time courses were detected.

## 2.1 Supplementary Figures


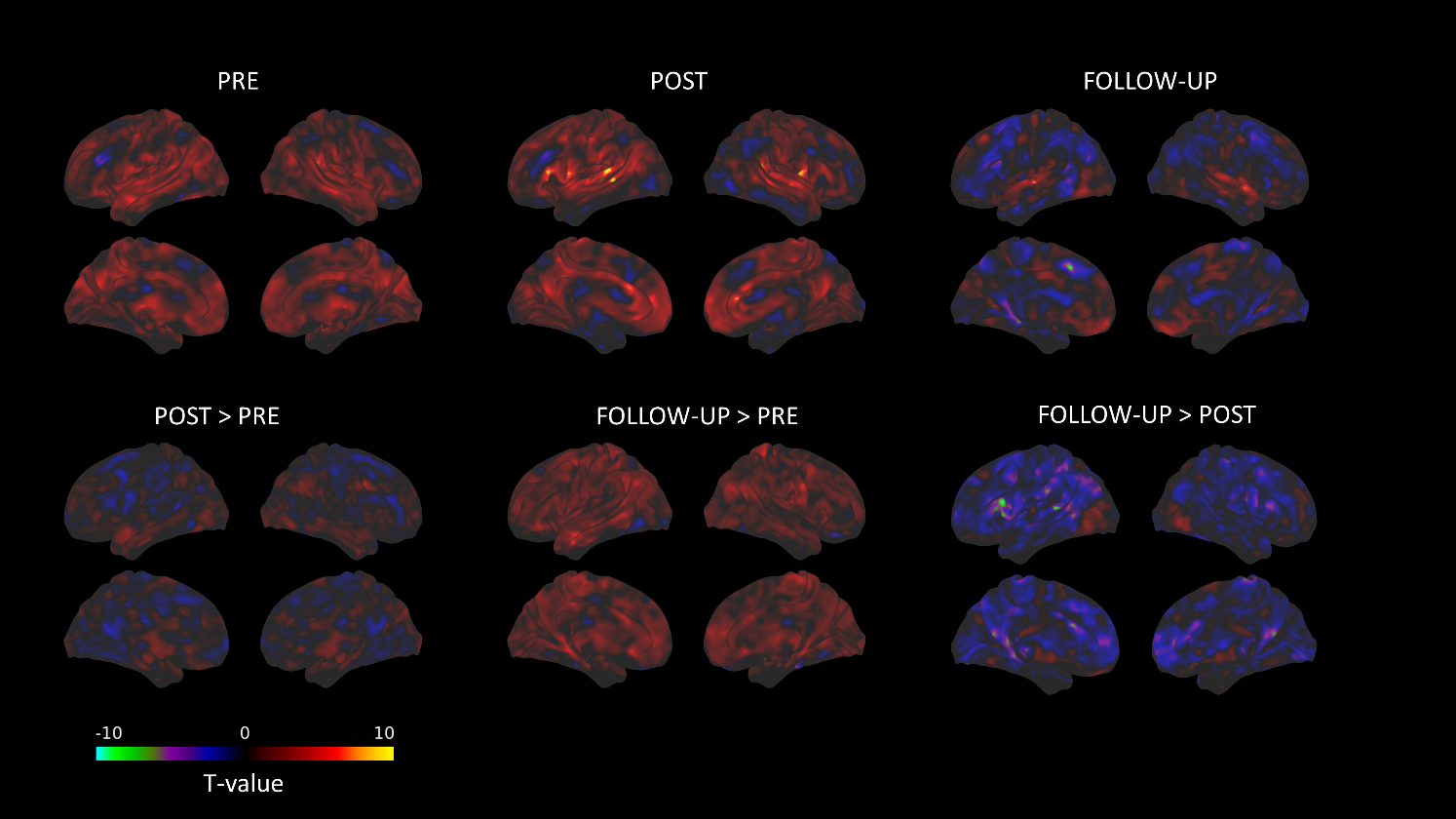
**Supplementary Figure 1.** Salient vs. neutral contrast in the affective sound categorization task within each session (top panel) and its difference between sessions (bottom panel).

**
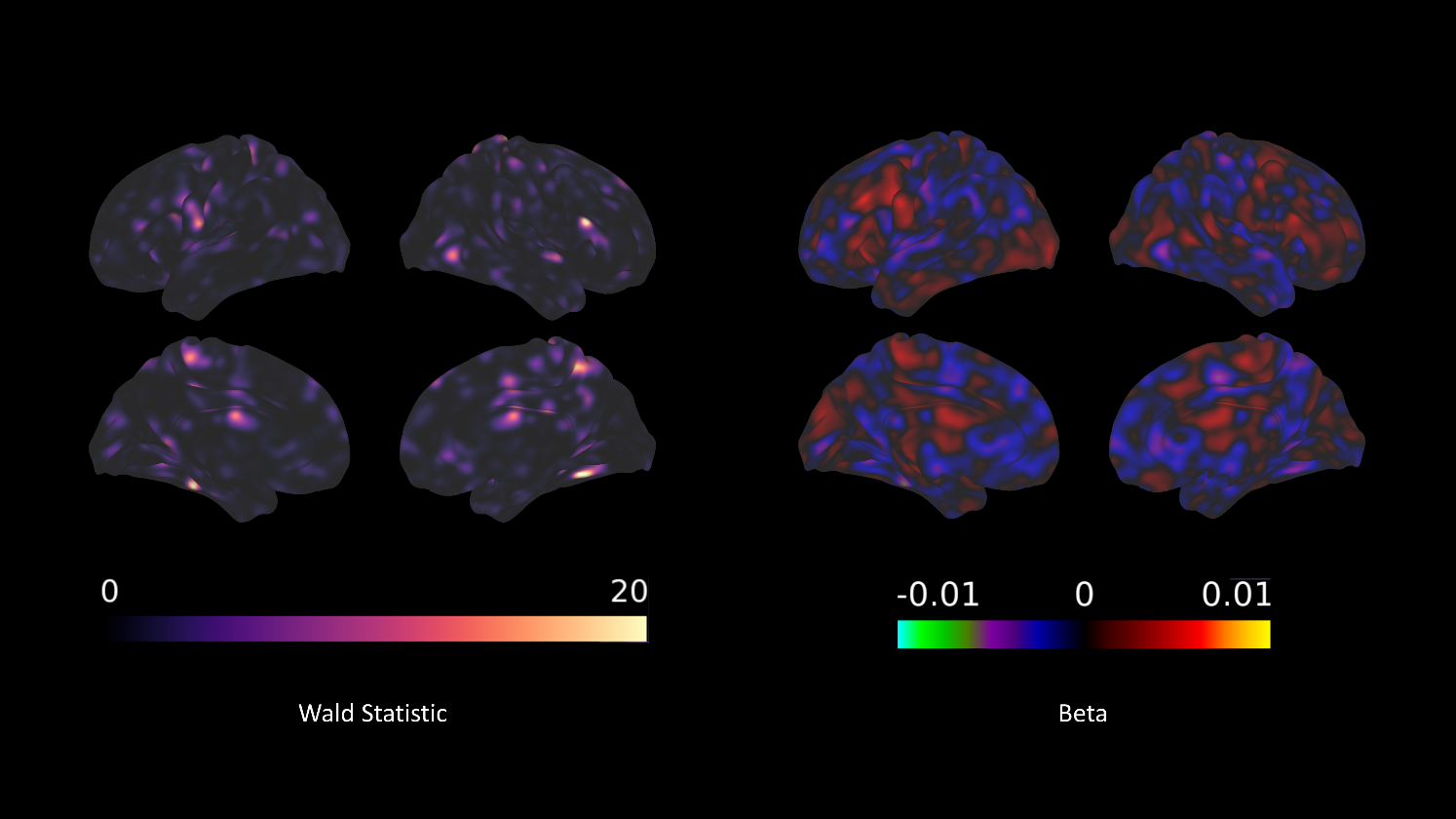
**

**Supplementary Figure 2.** Spatial map of how TFI predicts salient vs. neutral contrasts. The left panel shows the Wald statistic, while the right panel indicates the beta value and the direction of the relationship.


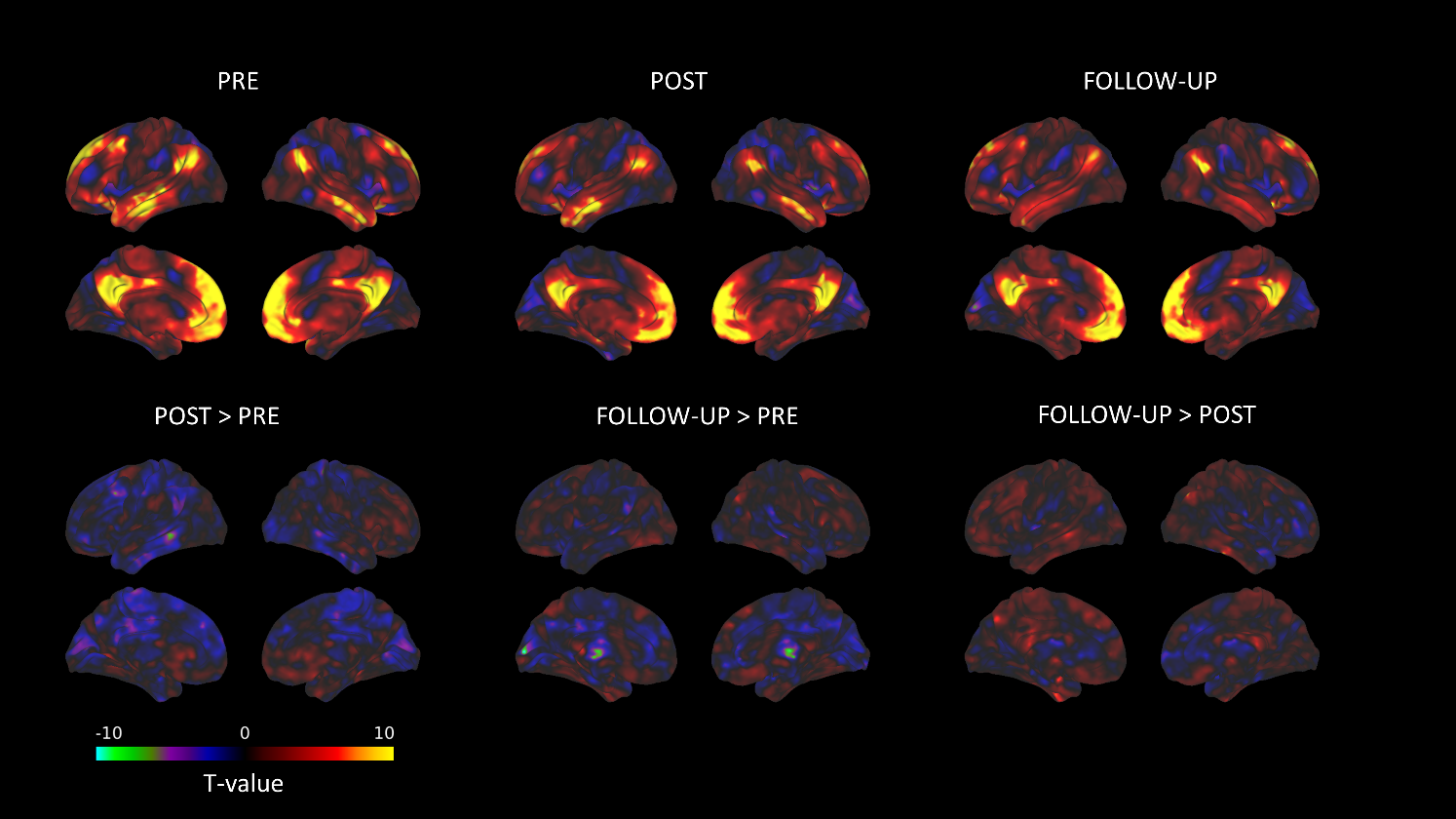


**Supplementary Figure 3.** Default mode network seed-voxel connectivity maps within each session (top panel) and the difference between sessions (bottom panel).


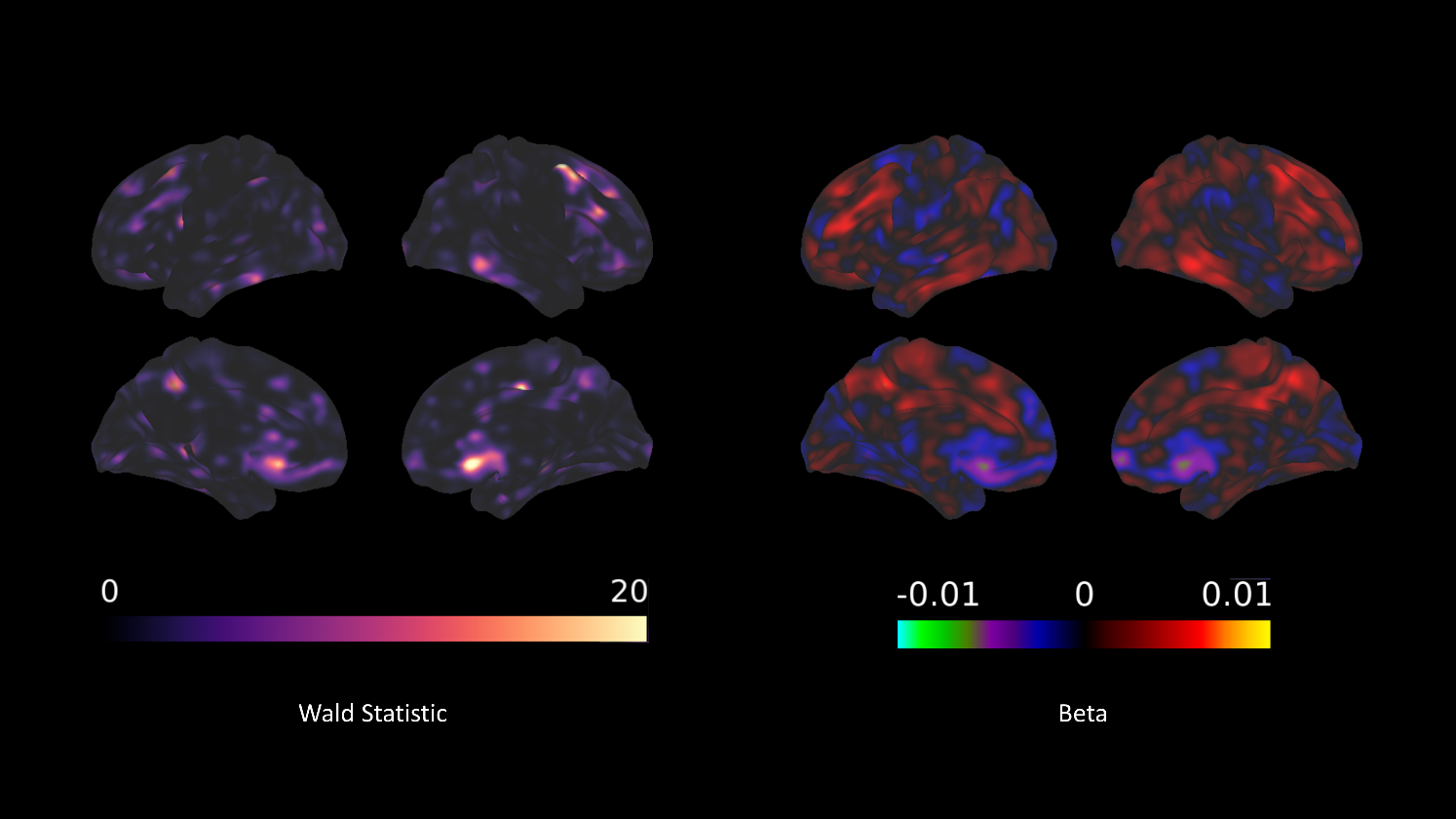


**Supplementary Figure 4.** Spatial map of how TFI predicts default mode network connectivity with the rest of the brain. The left panel shows the Wald statistic, while the right panel indicates the beta value and the direction of the relationship.


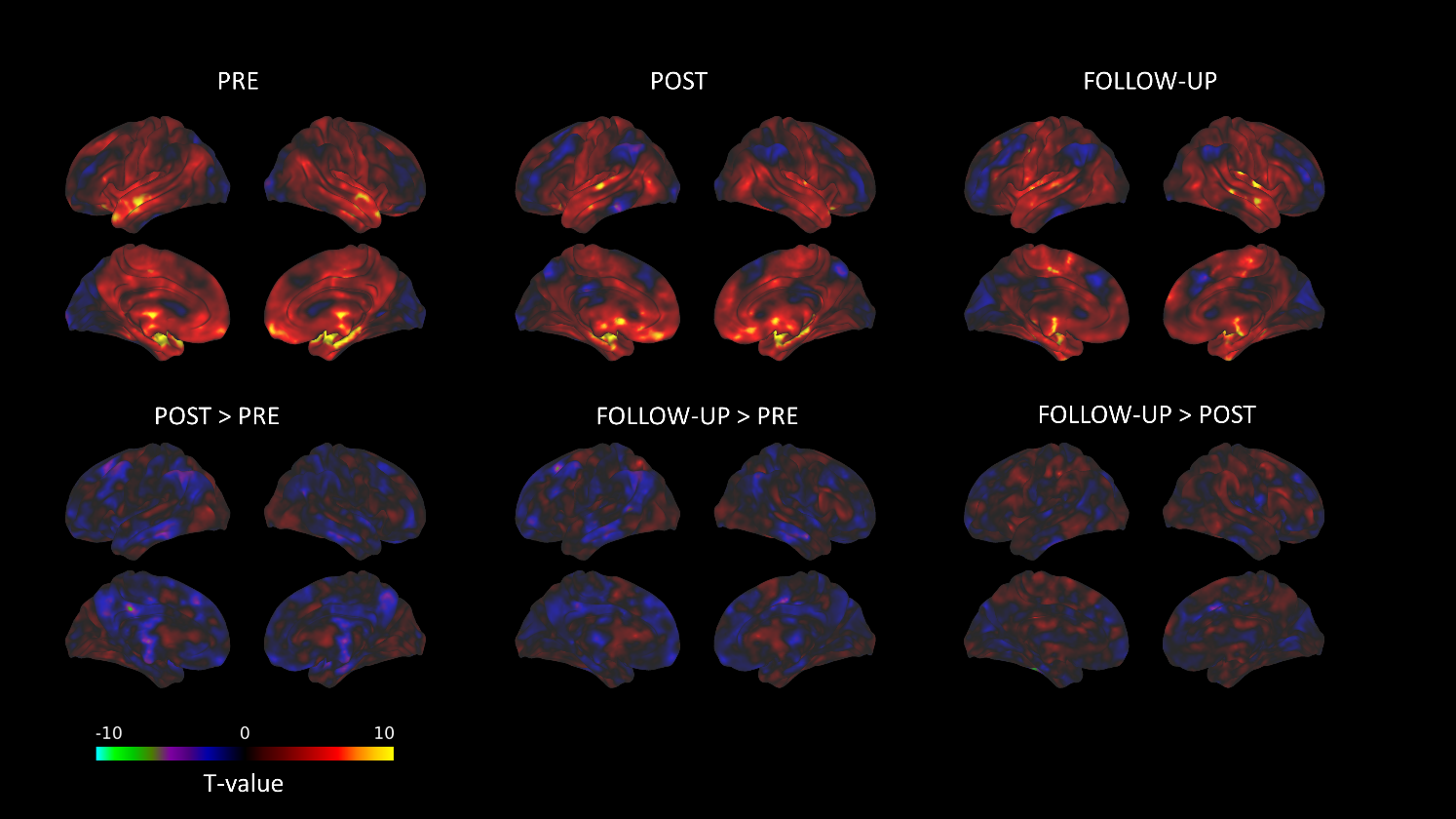
**Supplementary Figure 5.** Amygdala seed-voxel connectivity maps within each session (top panel) and the difference between sessions (bottom panel).

**
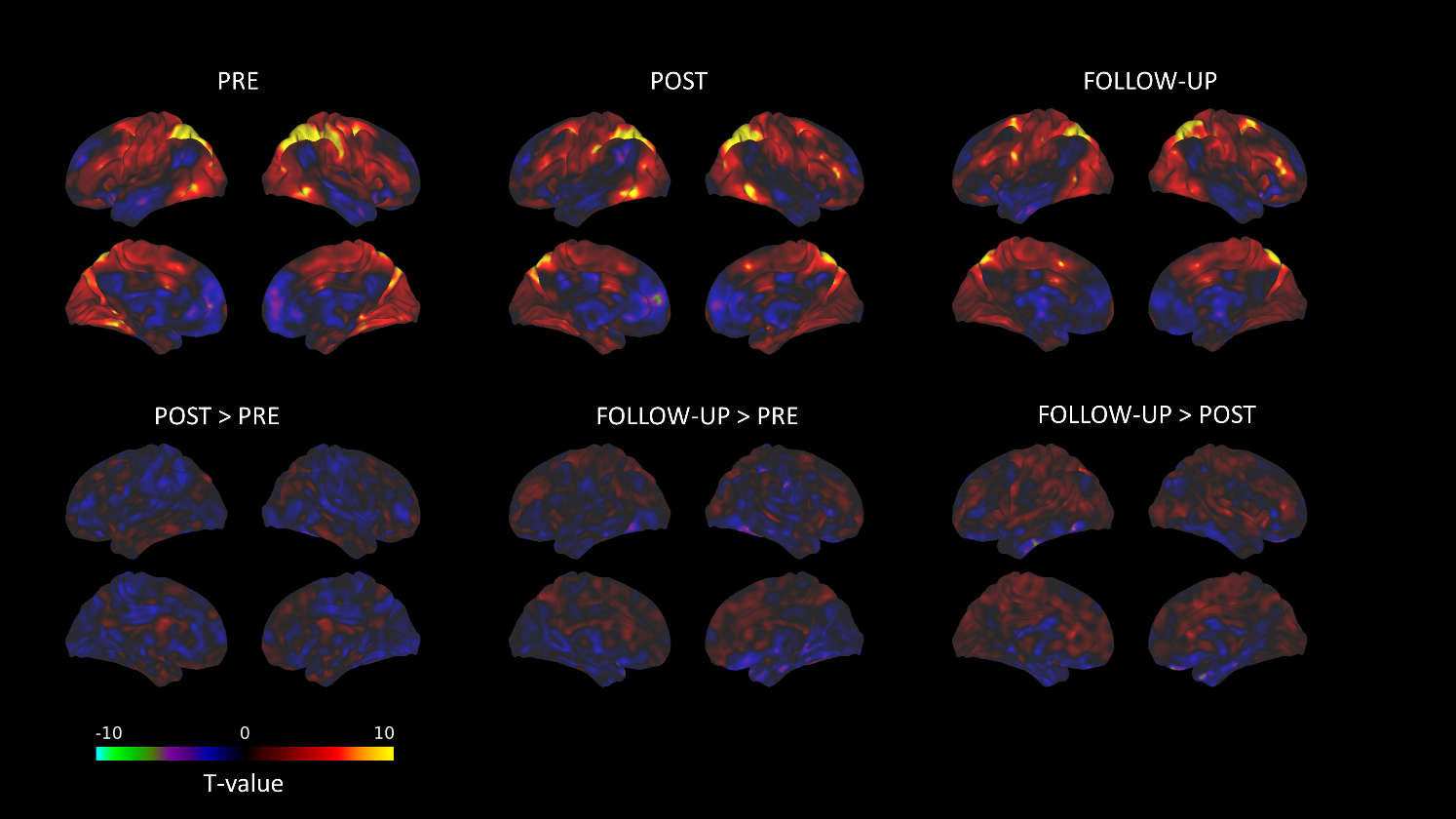
**

**Supplementary Figure 6.** Dorsal attention network (1) seed-voxel connectivity maps within each session (top panel) and the difference between sessions (bottom panel).


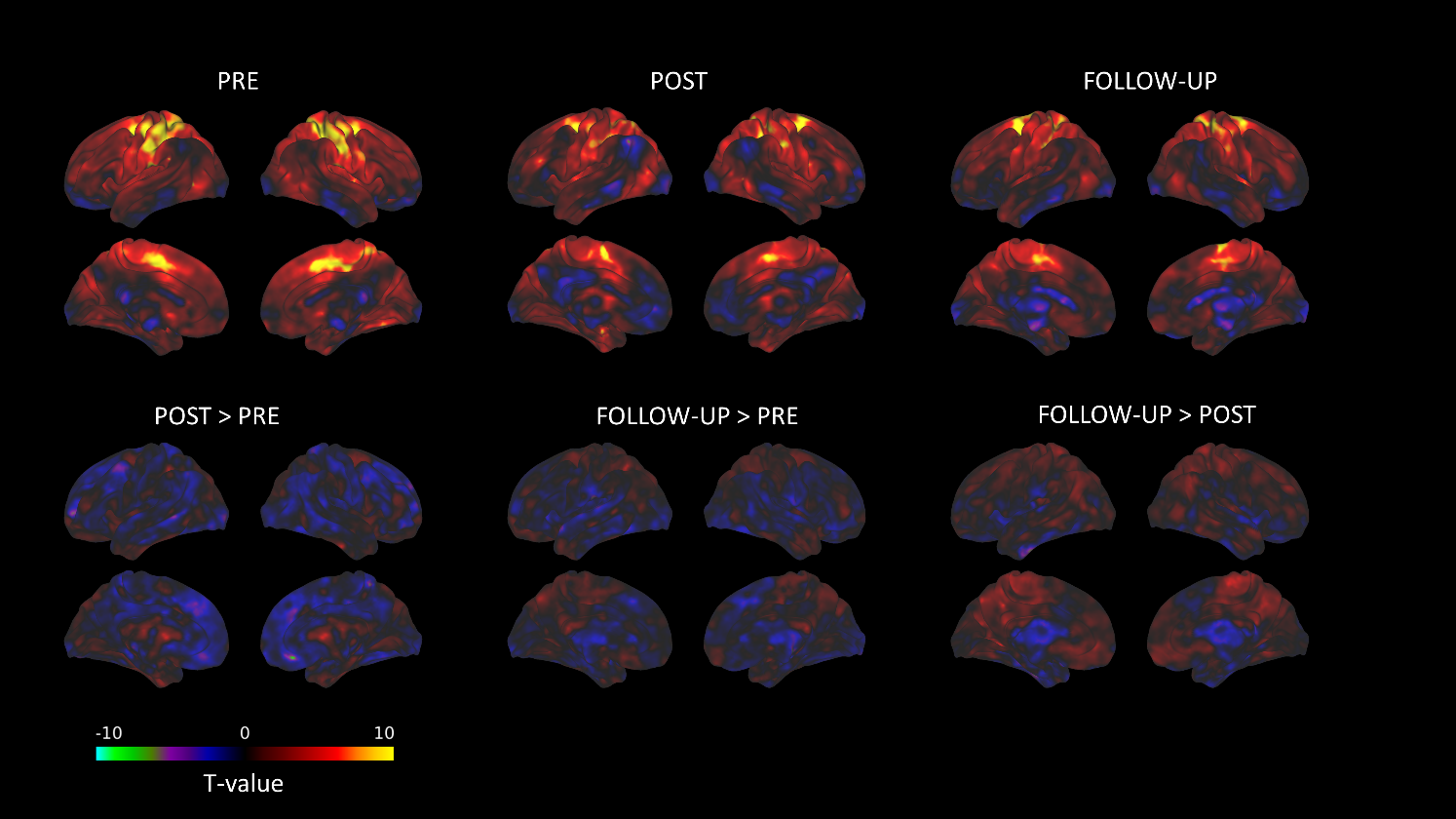


**Supplementary Figure 7.** Dorsal attention network (2) seed-voxel connectivity maps within each session (top panel) and the difference between sessions (bottom panel).


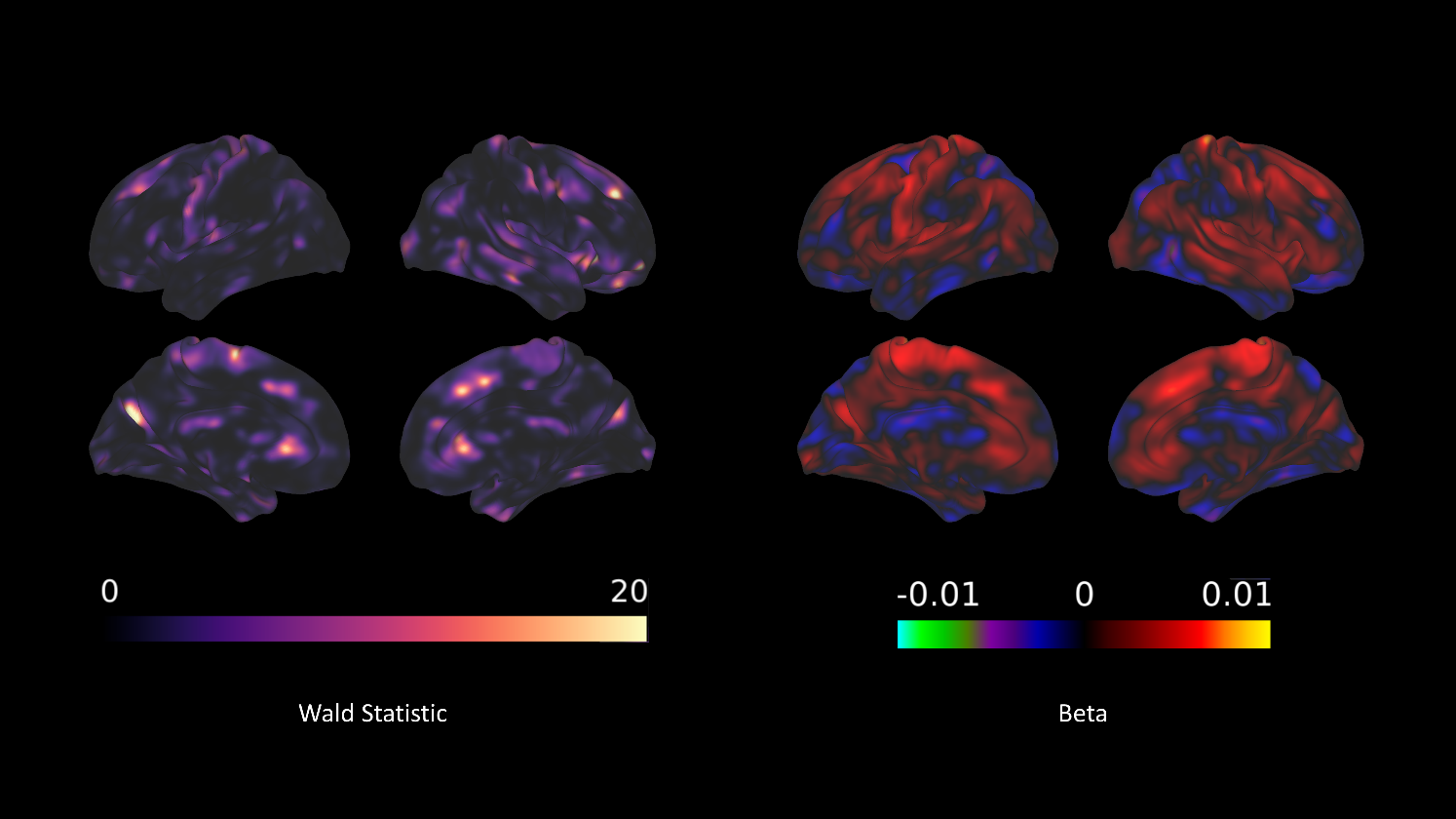


**Supplementary Figure 8.** Spatial map of how TFI predicts dorsal attention network (2) connectivity with the rest of the brain. The left panel shows the Wald statistic, while the right panel indicates the beta value and the direction of the relationship.


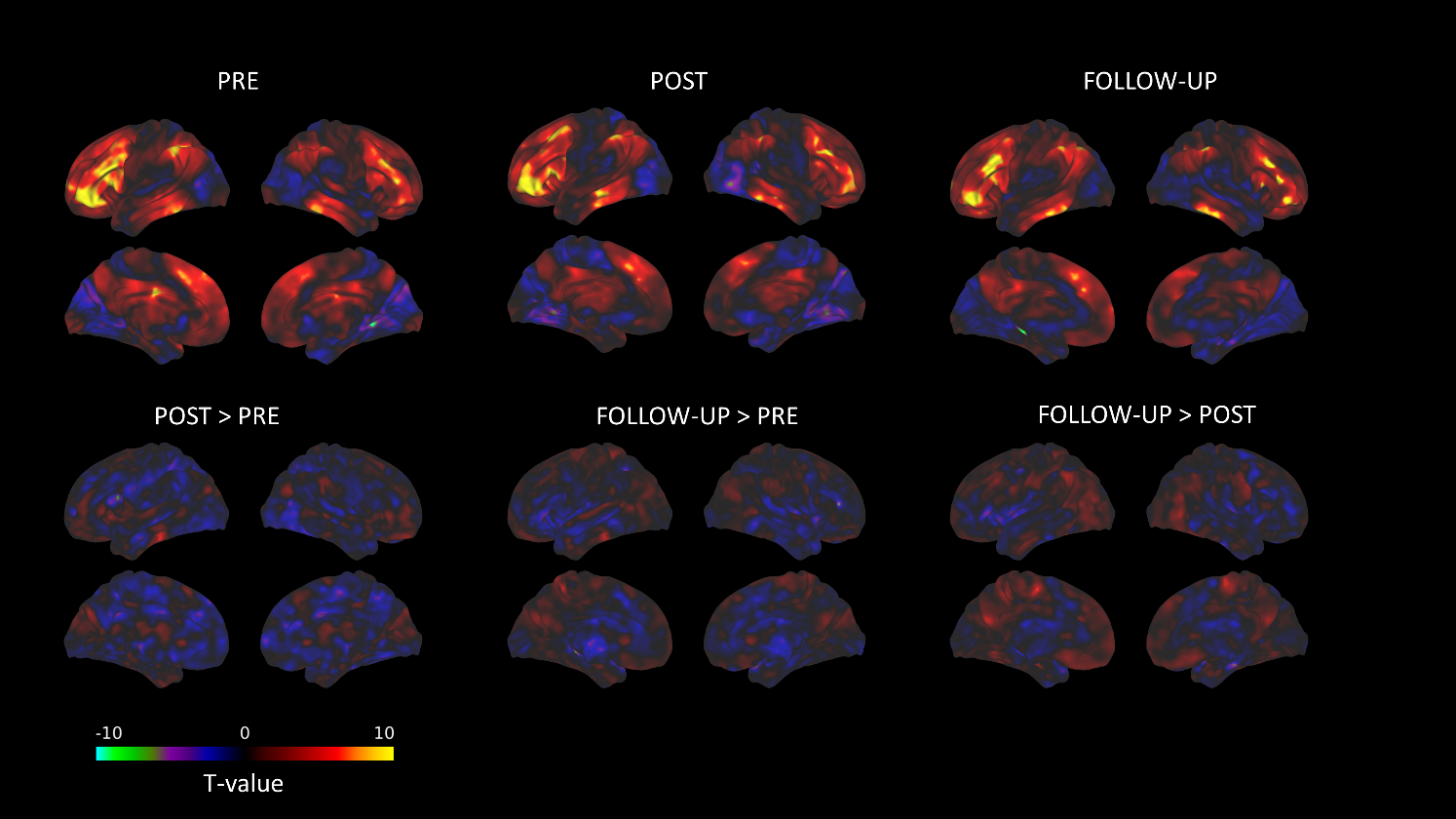


**Supplementary Figure 9.** Left fronto-parietal network seed-voxel connectivity maps within each session (top panel) and the difference between sessions (bottom panel).


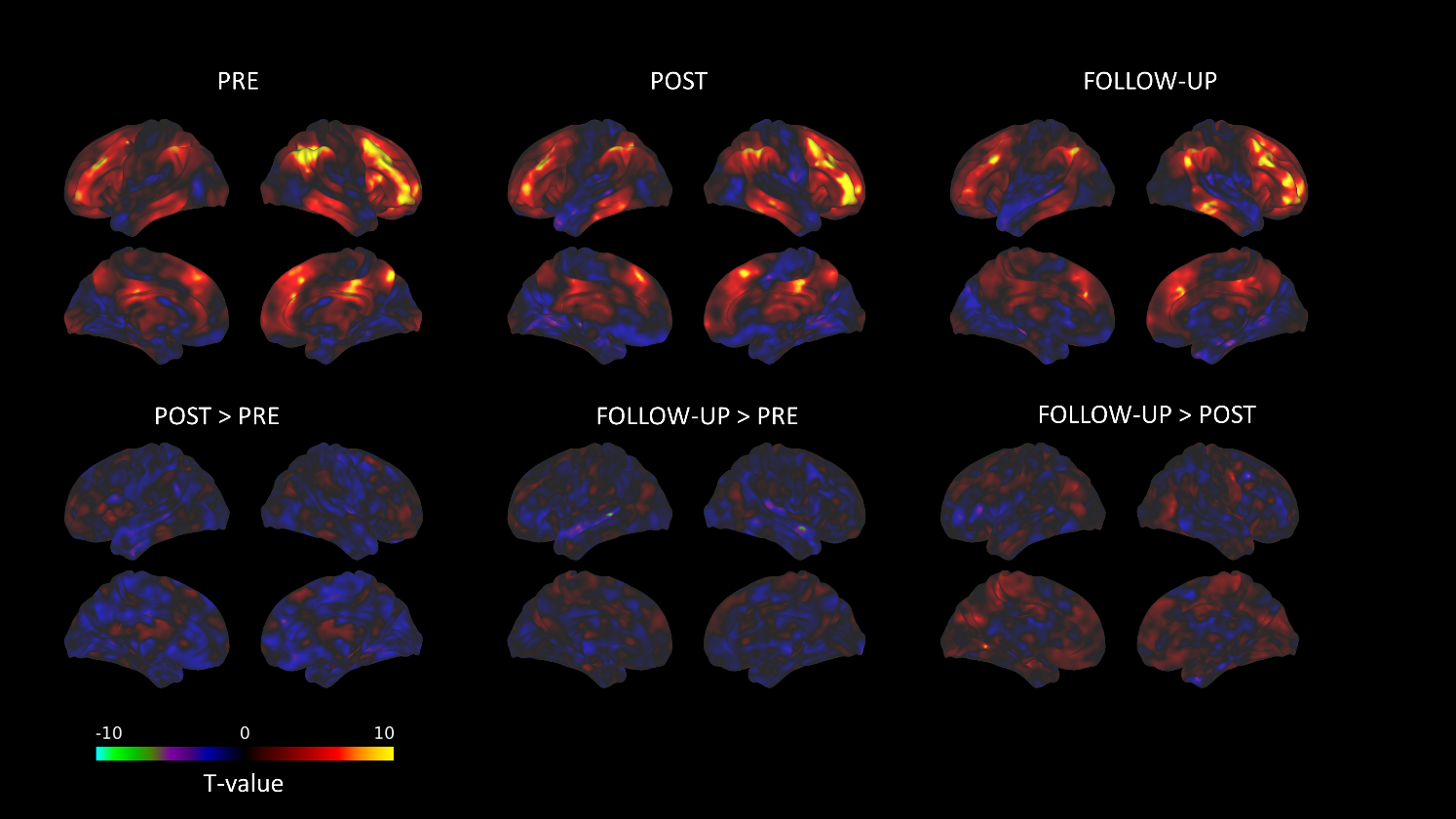


**Supplementary Figure 10.** Right fronto-parietal network seed-voxel connectivity maps within each session (top panel) and the difference between sessions (bottom panel).


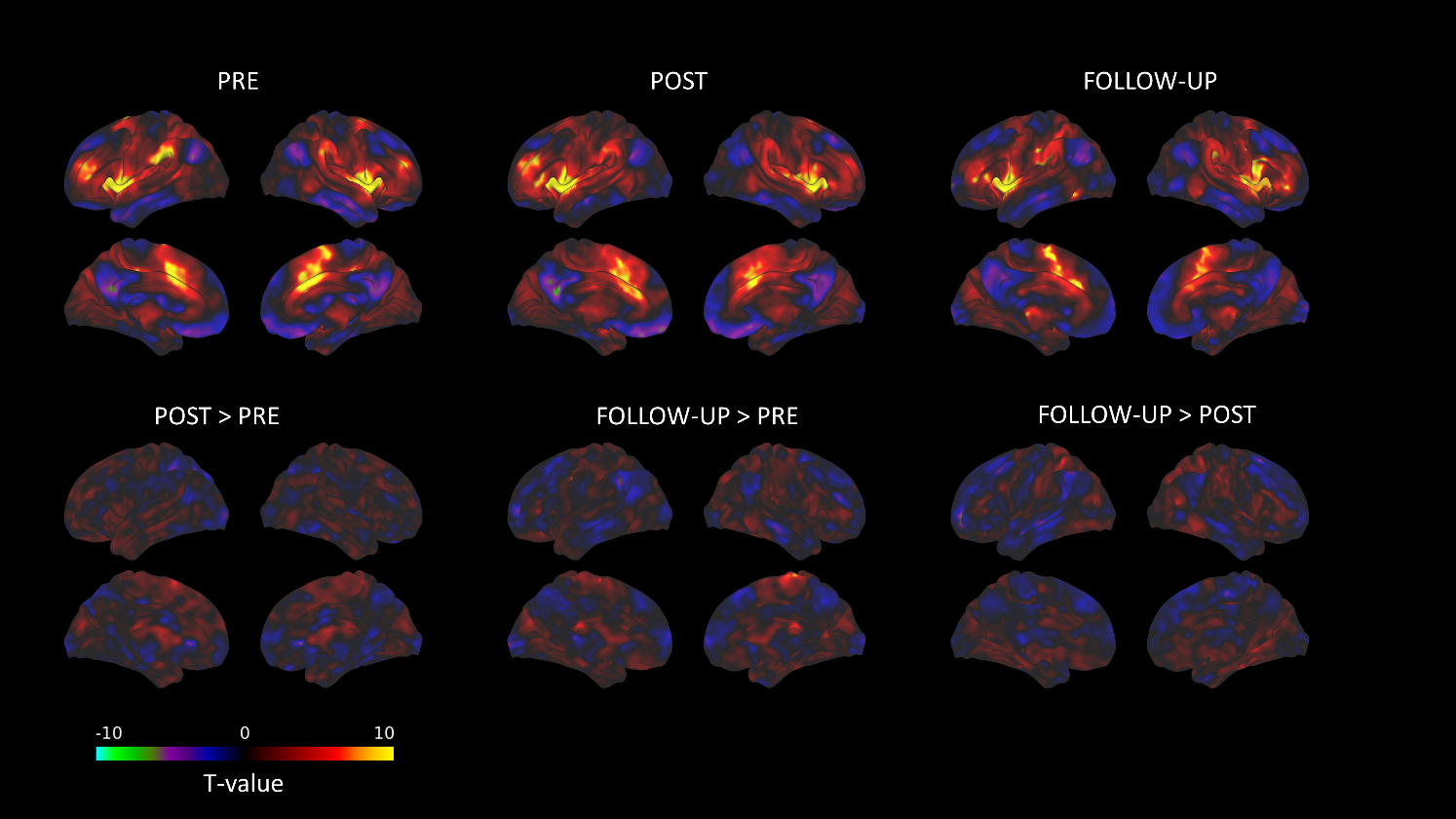


**Supplementary Figure 11.** Left cingulo-opercular network seed-voxel connectivity maps within each session (top panel) and the difference between sessions (bottom panel).


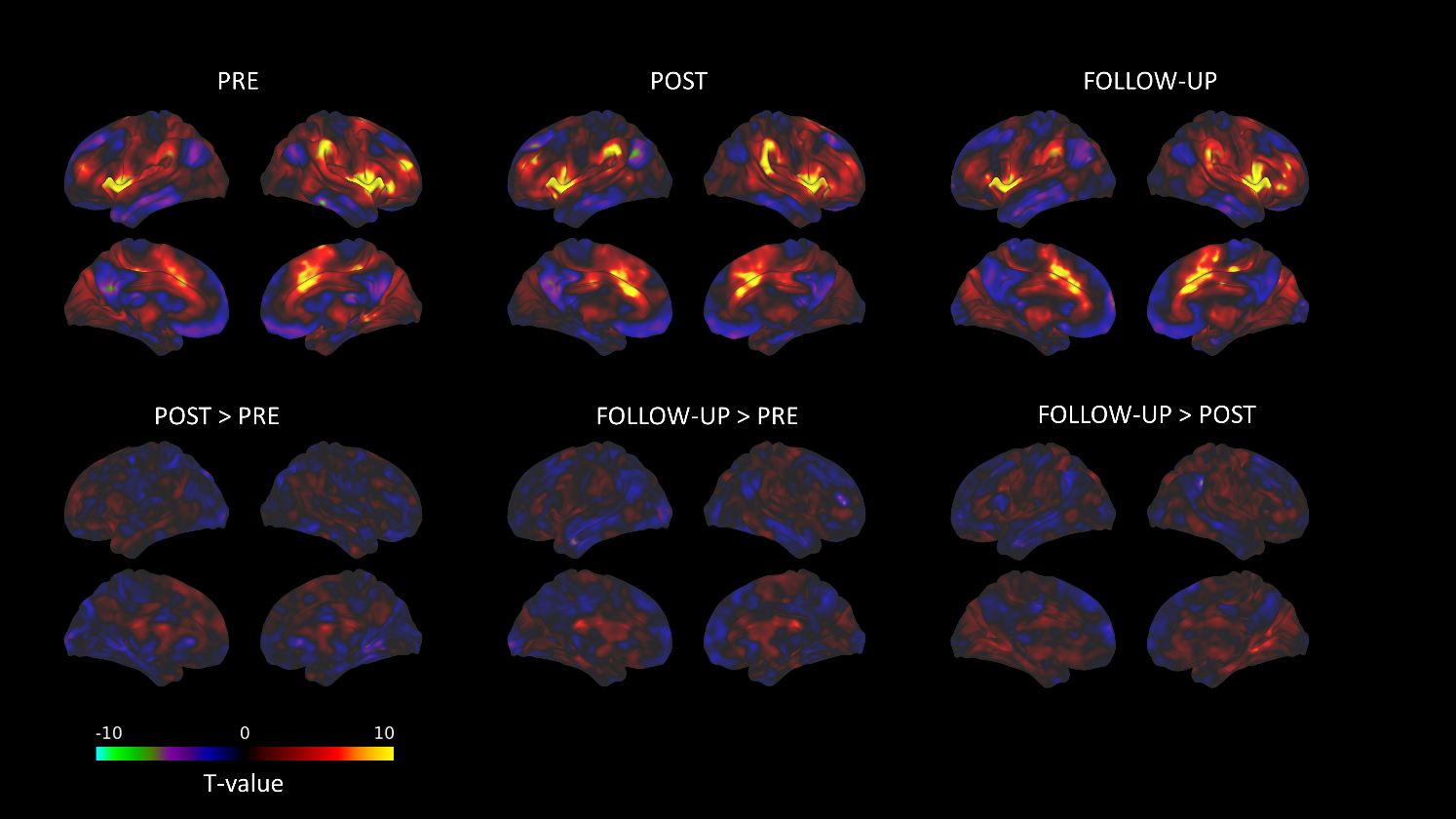


**Supplementary Figure 12.** Right cingulo-opercular network seed-voxel connectivity maps within each session (top panel) and the difference between sessions (bottom panel).


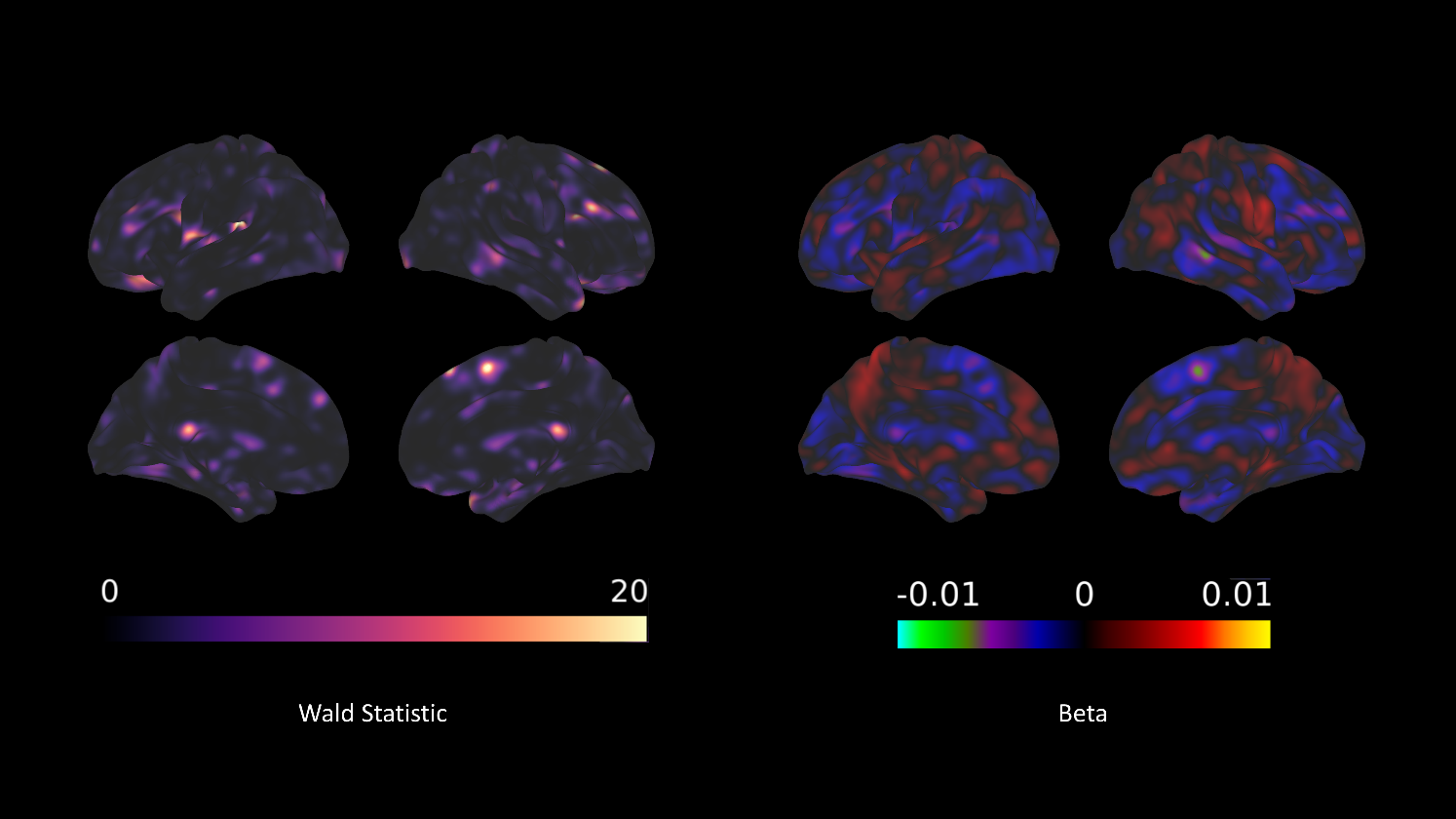


**Supplementary Figure 13.** Spatial map of how TFI predicts left cingulo-opercular network connectivity with the rest of the brain. The left panel shows the Wald statistic, while the right panel indicates the beta value and the direction of the relationship.


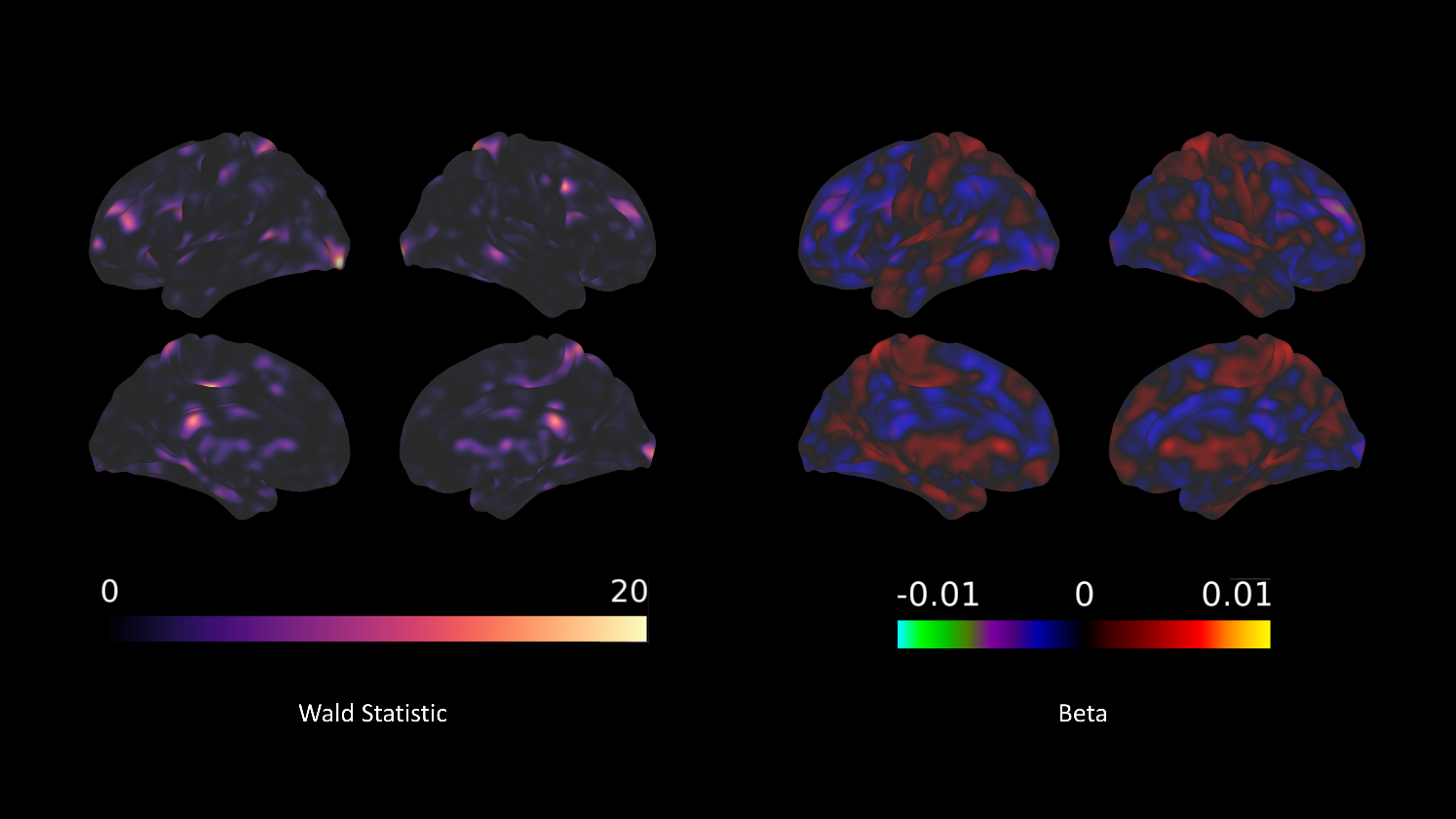


**Supplementary Figure 14.** Spatial map of how TFI predicts right cingulo-opercular connectivity with the rest of the brain. The left panel shows the Wald statistic, while the right panel indicates the beta value and the direction of the relationship.


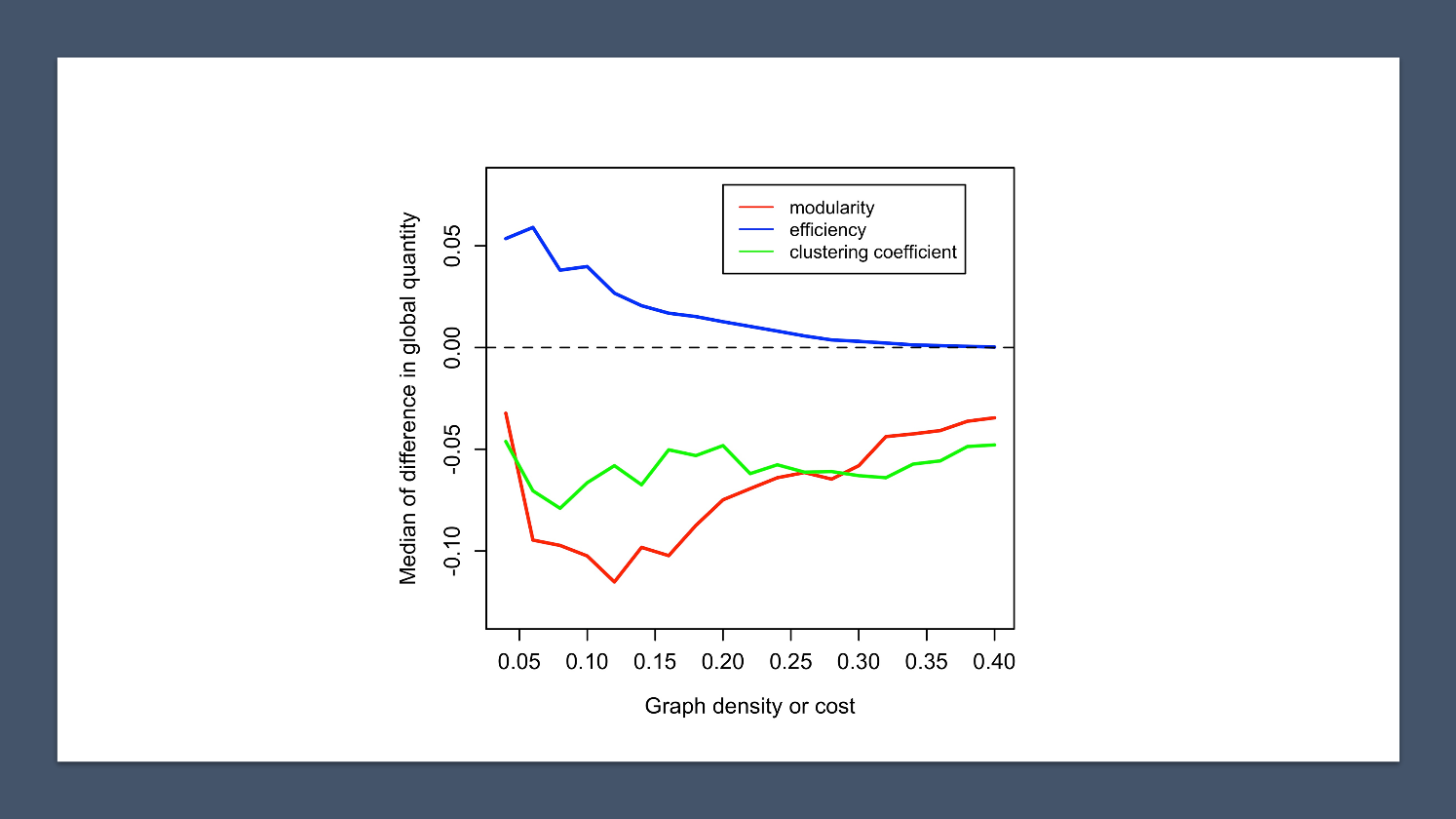


**Supplementary Figure 15.** Median of pre-post intervention differences in modularity, global efficiency, and global clustering across the subjects as a function of increasing network density.


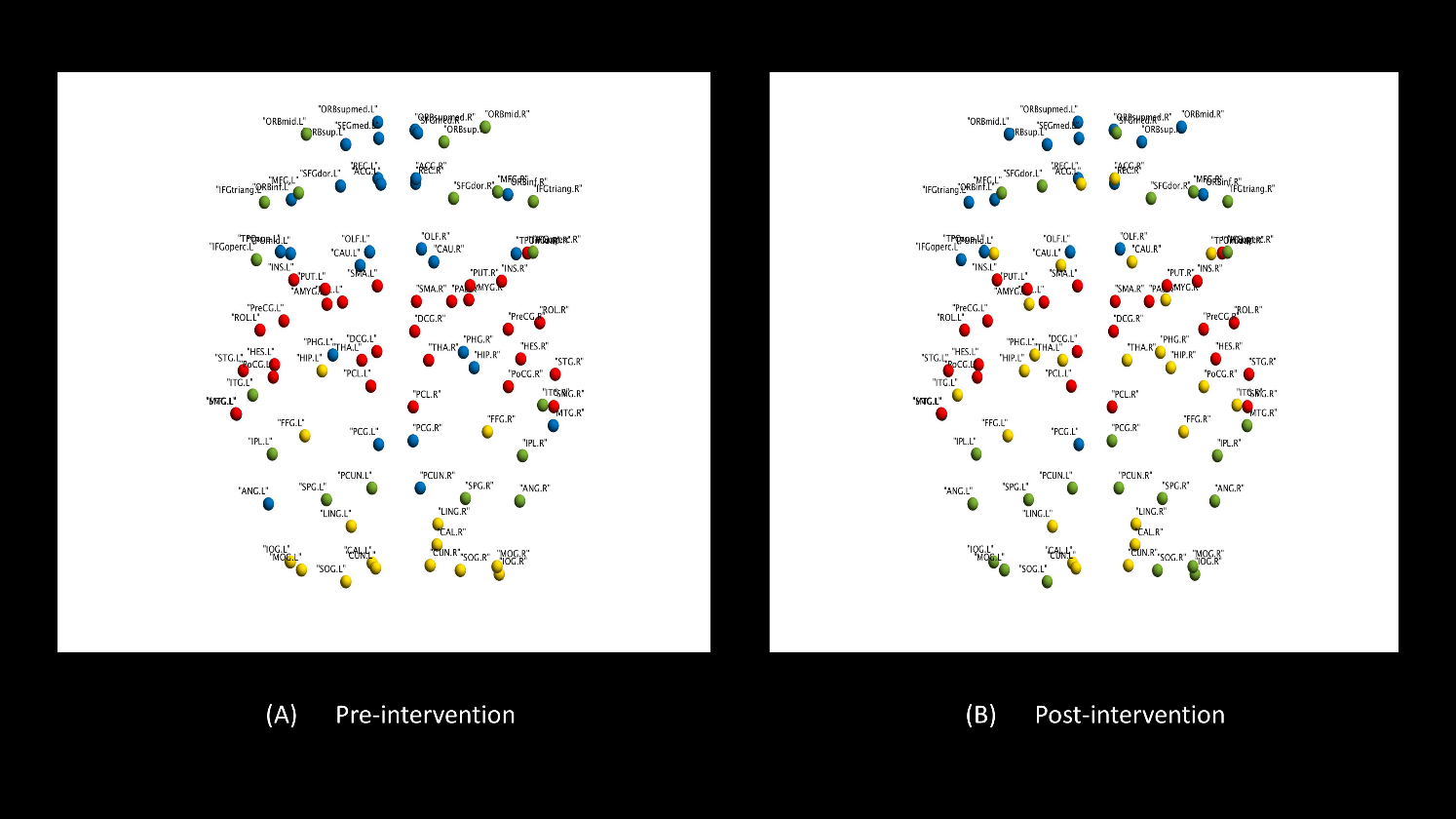


**Supplementary Figure 16.** Group putative community structure of the brain networks of the subjects in (a) pre-intervention and b) post-intervention. ROIs are colored according to their group putative community obtained from Co-OSNTF method applied to the group of networks with 0.20 connection density.


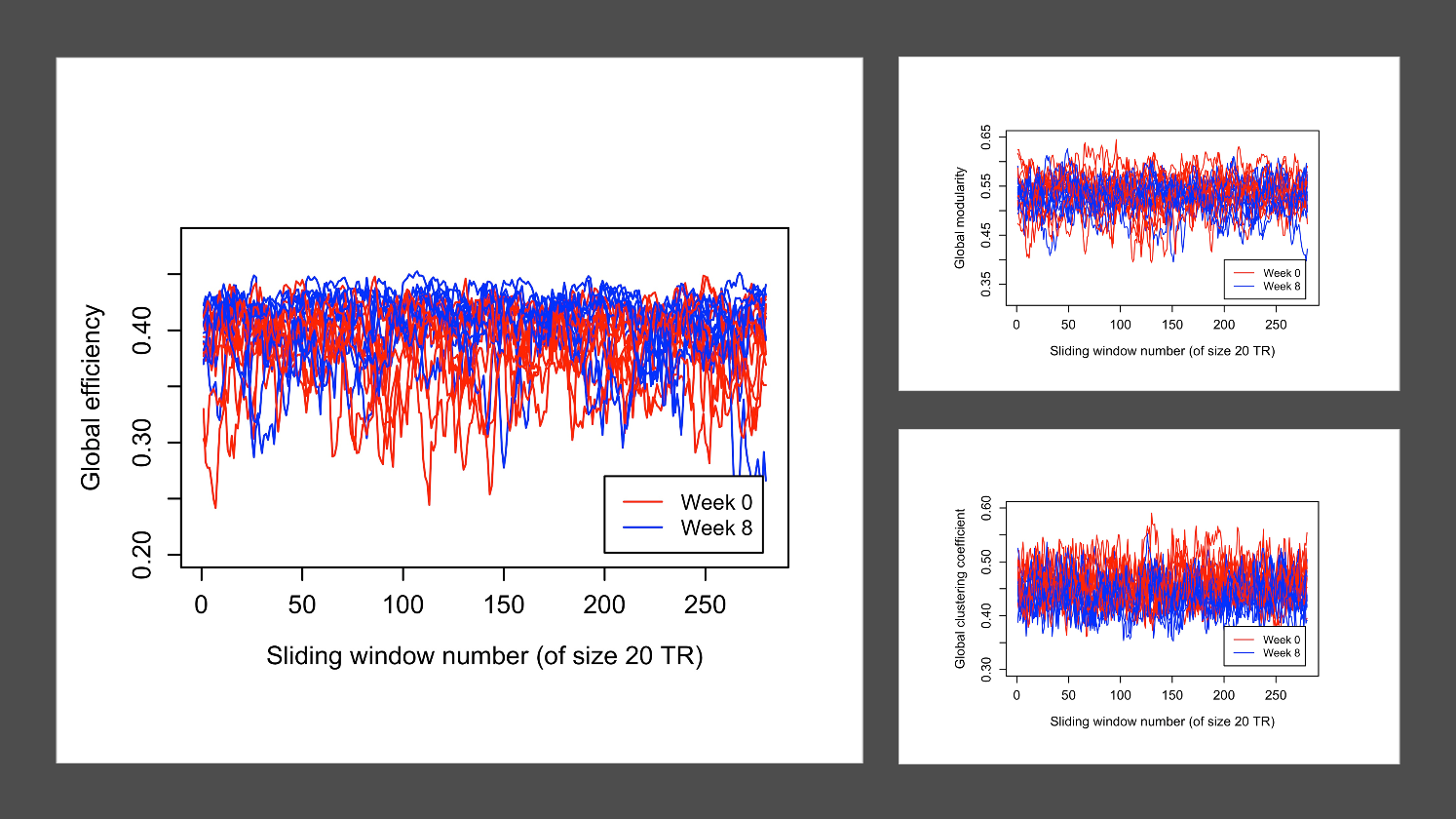


**Supplementary Figure 17.** Dynamic fluctuations of efficiency, modularity and clustering coefficient in the subject brain networks at pre and post intervention sessions. Note the time courses are not aligned among the subjects.


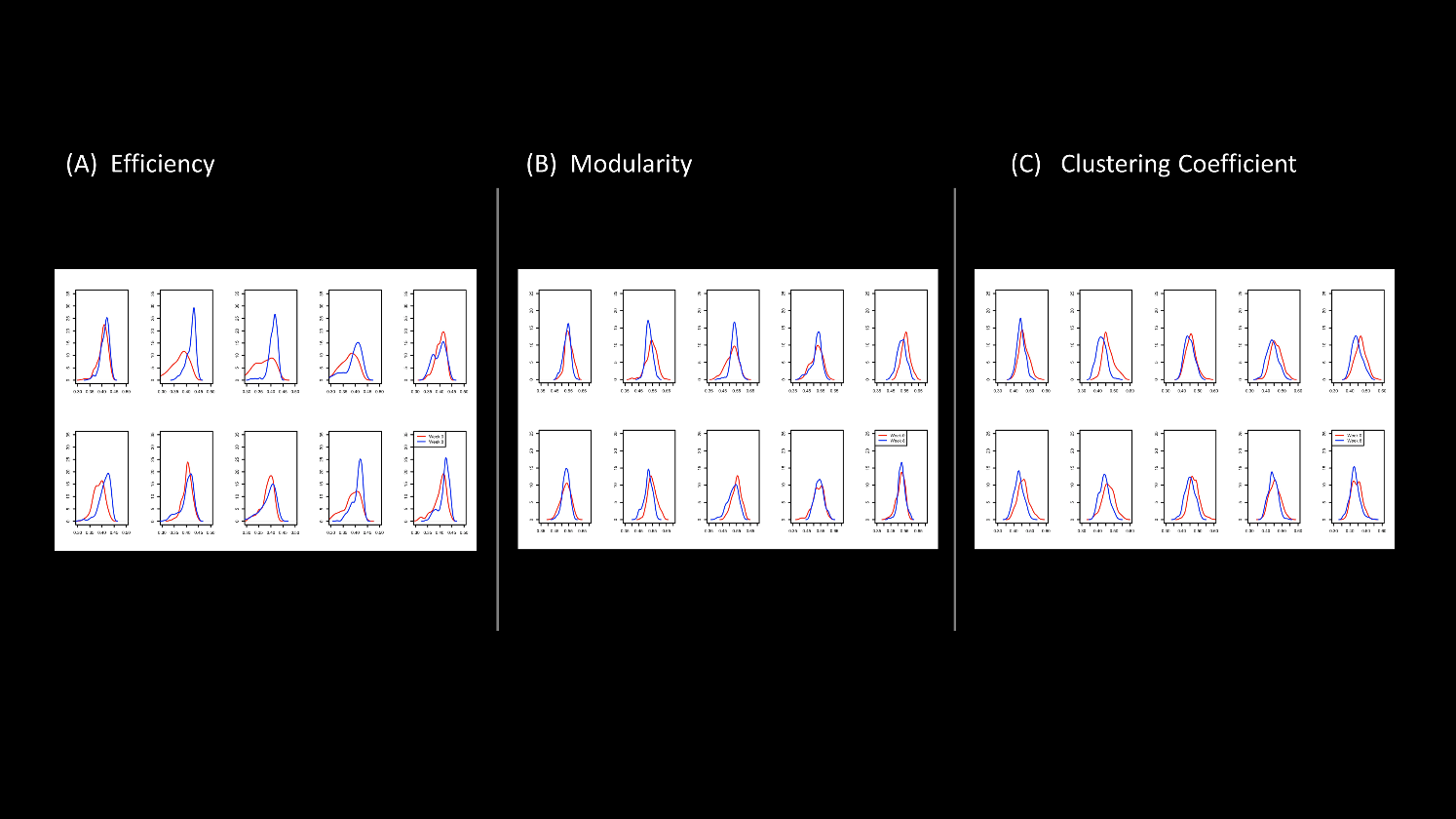


**Supplementary Figure 18.** Distribution of (A) efficiency, (B) modularity and (C) clustering coefficient for time varying brain networks over the entire sessions in all 10 subjects at pre and post intervention.

## 2.2 Supplementary Tables

**Supplementary Table 1**

*Summary sample screening demographics from the samples included at post and follow-up sessions*

| **Post-intervention** |  |  |  |  |
| --- | --- | --- | --- | --- |
| Gender | M = 4; F = 6 |  |  |  |
|  |  |  |  |  |
|  | Mean | SD |  |  |
| Age (year) | 52.40 | 10.35 |  |  |
| TFI | 35.40 | 15.80 |  |  |
| BDI | 10.50 | 9.05 |  |  |
| BAI | 7.50 | 7.47 |  |  |
|  |  |  |  |  |
| **Follow-up** |  |  |  |  |
| Gender | M = 4; F = 4 |  |  |  |
|  |  |  |  |  |
|  | Mean | SD |  |  |
| Age (year) | 51.75 | 11.59 |  |  |
| TFI | 35.38 | 21.07 |  |  |
| BDI | 9.38 | 7.96 |  |  |
| BAI | 7.38 | 7.60 |  |  |
|  |  |  |  |  |

**Supplementary Table 2**

*Affective sound presentation order for each MRI session*

| Session 1 |  | Session 2 |  | Session 3 |  |
| --- | --- | --- | --- | --- | --- |
| Sound | IADS ID | Sound | IADS ID | Sound | IADS ID |
|  |  |  |  |  |  |
| 'Hiccup' | 245 | 'Cattle' | 114 | 'Guitar' | 816 |
| 'Clock' | 708 | 'AlarmClock' | 709 | 'CarHorns' | 420 |
| 'RockNRoll' | 815 | 'SportsCrowd' | 352 | 'BikeWreck' | 600 |
| 'BabyCry' | 261 | 'Paper1' | 728 | 'PlaneCrash' | 501 |
| 'MaleLaugh' | 221 | 'HorseRace' | 363 | 'Crowd5' | 368 |
| 'Creep' | 288 | 'Cattle' | 114 | 'Bugle' | 808 |
| 'Clock' | 708 | 'Seagull' | 150 | 'Wind' | 500 |
| 'Shower' | 206 | 'FemScream3' | 277 | 'Explosion' | 626 |
| 'RockNRoll' | 815 | 'Giggling' | 230 | 'FunkMusic' | 820 |
| 'Victim' | 286 | 'Shovel' | 382 | 'Fan' | 701 |
| 'HeartBeat' | 246 | 'Whistling' | 270 | 'Bugle' | 808 |
| 'Party' | 365 | 'Belch' | 702 | 'Rain1' | 627 |
| 'Toilet' | 700 | 'Robin' | 151 | 'EroticFem2' | 202 |
| 'Buzzing' | 116 | 'Radio' | 723 | 'Baseball' | 353 |
| 'ChildAbuse' | 278 | 'Fight2' | 282 | 'Growl2' | 133 |
| 'MaleScream' | 292 | 'Bees' | 115 | 'MusicBox' | 111 |
| 'Chewing' | 724 | 'TireSkids' | 422 | 'Rain1' | 627 |
| 'Toilet' | 700 | 'SlotMachine1' | 716 | 'Office 2' | 319 |
| 'ClapGame' | 225 | 'EroticFem1' | 201 | 'Buzzer' | 712 |
| 'Attack3' | 284 | 'Sink' | 374 | 'Sirens' | 713 |
| 'CountryNight' | 171 | 'Bees' | 115 | 'GunShot' | 289 |
| 'Casino2' | 367 | 'Prowler' | 291 | 'Rain1' | 627 |
| 'Bomb' | 699 | 'MaleCough' | 241 | 'Fan' | 701 |
| 'Cat' | 102 | 'Growl1' | 106 | 'ColonialMusic' | 601 |
| 'Hiccup' | 245 | 'Bar' | 364 | 'TypeWriter' | 322 |
| 'MaleScream' | 292 | 'SlotMachine1' | 716 | 'Casino1' | 366 |
| 'Cows' | 113 | 'Prowler' | 291 | 'EroticFem2' | 202 |
| 'Vomit' | 255 | 'Night' | 170 | 'Injury' | 423 |
| 'Bomb' | 699 | 'Kids1' | 112 | 'Train' | 425 |
| 'ChildAbuse' | 278 | 'SlotMachine2' | 717 | 'Beer' | 721 |
| 'Kids2' | 224 | 'EroticFem3' | 205 | 'Casino1' | 366 |
| 'Rain1' | 377 | 'Fight2' | 282 | 'Baby' | 110 |
| 'HeartBeat' | 246 | 'Crowd2' | 311 | 'Paint' | 373 |
| 'Crowd4' | 355 | 'Rooster' | 120 | 'Crowd3' | 312 |
| 'ManWheeze' | 244 | 'Dog' | 107 | 'Guitar' | 816 |
| 'Walking' | 722 | 'EroticFem1' | 201 | 'Laughing' | 226 |
| 'Cows' | 113 | 'Kids1' | 112 | 'Wedding' | 813 |
| 'Applause1' | 351 | 'Paper1' | 728 | 'Puppy' | 105 |
| 'Chickens' | 132 | 'BabiesCry' | 260 | 'Paint' | 373 |
| 'ManWheeze' | 244 | 'AirRaid' | 624 | 'Sirens' | 713 |
| 'RollerCoaster' | 360 | 'ManSobbing' | 293 | 'Countdown' | 415 |
| 'Fight1' | 290 | 'Tropical' | 152 | 'Train' | 425 |
| 'Crowd4' | 355 | 'Siren2' | 714 | 'Bach' | 811 |
| 'EroticCouple2' | 215 | 'Rooster' | 120 | 'Crowd3' | 312 |
| 'Attack3' | 284 | 'AlarmClock' | 709 | 'Baseball' | 353 |
| 'ClapGame' | 225 | 'Paper2' | 729 | 'Lawnmower' | 376 |
| 'EroticCouple' | 200 | 'SportsCrowd' | 352 | 'Beer' | 721 |
| 'Phone1' | 704 | 'HorseRace' | 363 | 'Wedding' | 813 |
| 'Party' | 365 | 'Helicopter2' | 410 | 'Pig' | 130 |
| 'DentistDrill' | 719 | 'Doorbell' | 378 | 'Panting' | 104 |
| 'Vomit' | 255 | 'Paper2' | 729 | 'Puppy' | 105 |
| 'BoyLaugh' | 220 | 'Carousel' | 109 | 'GunShot' | 289 |
| 'Casino2' | 367 | 'Tropical' | 152 | 'Lawnmower' | 376 |
| 'Siren1' | 711 | 'EroticFem3' | 205 | 'Helicopter1' | 403 |
| 'Phone1' | 704 | 'SlotMachine2' | 717 | 'Countdown' | 415 |
| 'Office1' | 320 | 'FemScream3' | 277 | 'Baby' | 110 |
| 'BabyCry' | 261 | 'Carousel' | 109 | 'CarHorns' | 420 |
| 'Writing' | 358 | 'Bar' | 364 | 'Growl2' | 133 |
| 'CountryNight' | 171 | 'Helicopter2' | 410 | 'Bach' | 811 |
| 'Shower' | 206 | 'Bongos' | 817 | 'MaleSnore' | 252 |
| 'Office1' | 320 | 'Attack1' | 279 | 'MusicBox' | 111 |
| 'Fight1' | 290 | 'Attack3' | 281 | 'Explosion' | 626 |
| 'Creep' | 288 | 'TireSkids' | 422 | 'MayDay' | 625 |
| 'Crowd1' | 310 | 'Seagull' | 150 | 'NativeSong' | 802 |
| 'Polaroid' | 375 | 'BabiesCry' | 260 | 'MaleSnore' | 252 |
| 'Writing' | 358 | 'Attack1' | 279 | 'Alarm' | 715 |
| 'EroticCouple' | 200 | 'Bongos' | 817 | 'Laughing' | 226 |
| 'DentistDrill' | 719 | 'Crash' | 732 | 'Injury' | 423 |
| 'Siren1' | 711 | 'AirRaid' | 624 | 'Scream' | 275 |
| 'BoyLaugh' | 220 | 'Whistling' | 270 | 'Rain1' | 627 |
| 'BrushTeeth' | 720 | 'Doorbell' | 378 | 'PlaneCrash' | 501 |
| 'RollerCoaster' | 360 | 'Shovel' | 382 | 'RattleSnake' | 134 |
| 'Cat' | 102 | 'Radio' | 723 | 'MayDay' | 625 |
| 'FemaleCough' | 242 | 'Night' | 170 | 'Scream' | 275 |
| 'Polaroid' | 375 | 'MaleCough' | 241 | 'Office 2' | 319 |
| 'Restaurant' | 361 | 'VideoGame' | 254 | 'Panting' | 104 |
| 'Rain1' | 377 | 'Attack3' | 281 | 'RattleSnake' | 134 |
| 'BrushTeeth' | 720 | 'Robin' | 151 | 'BikeWreck' | 600 |
| 'Applause1' | 351 | 'Crash' | 732 | 'Yawn' | 262 |
| 'Chewing' | 724 | 'Dog' | 107 | 'Wind' | 500 |
| 'Restaurant' | 361 | 'VideoGame' | 254 | 'TypeWriter' | 322 |
| 'Walking' | 722 | 'Carousel' | 109 | 'ColonialMusic' | 601 |
| 'FemaleCough' | 242 | 'Siren2' | 714 | 'Buzzer' | 712 |
| 'Victim' | 286 | 'Growl1' | 106 | 'NativeSong' | 802 |
| 'Buzzing' | 116 | 'Giggling' | 230 | 'Helicopter1' | 403 |
| 'Crowd1' | 310 | 'Crowd2' | 311 | 'Alarm' | 715 |
| 'Kids2' | 224 | 'Carousel' | 109 | 'Pig' | 130 |
| 'Chickens' | 132 | 'ManSobbing' | 293 | 'Yawn' | 262 |
| 'EroticCouple2' | 215 | 'Sink' | 374 | 'Crowd5' | 368 |
| 'MaleLaugh' | 221 | 'Belch' | 702 | 'FunkMusic' | 820 |

**Supplementary Table 3**

*Correlation of normalized change in TFI score with normalized changes in modularity and clustering coefficient*

| **Graph Density** | **Modularity (p value)** | **Clustering coefficient (p value)** |
| --- | --- | --- |
| 0.05 | 0.4590 (0.0736) | 0.4978 (0.0497*) |
| 0.10 | 0.5090 (0.0440*) | 0.5187 (0.0395*) |
| 0.15 | 0.3692 (0.1593) | 0.3625 (0.1675) |
| 0.20 | 0.4358 (0.0915) | 0.3815 (0.1447) |
| 0.25 | 0.4296 (0.0967) | 0.3688 (0.1597) |

** *p* < .01 levels (*p* < .05, FWER level), * *p* < .05

**Supplementary Table 4**

*Regressing TFI score differences with the differences in measures of functional segregation.*

| **Graph Density** | $\boldsymbol{R}^{\boldsymbol{2}}$ | **F test *p*-value** |
| --- | --- | --- |
| 0.05 | 0.5487 | 0.0057** |
| 0.10 | 0.4568 | 0.0189* |
| 0.15 | 0.2452 | 0.1607 |
| 0.20 | 0.3308 | 0.0734 |
| 0.25 | 0.3235 | 0.0788 |

** *p* < .01 levels (*p* < .05, FWER level), * *p* < .05.

**Supplementary Table 5**

*Paired two sample tests between pre and post intervention subject brain networks in terms of the three global network summary measures.*

| **Graph Density** | **Paired t-test (*p-value*)** | **Non-parametric test (*p-value*)** |
| --- | --- | --- |
| *Efficiency* |  |  |
| 0.05 | 0.0044** | 0.0097** |
| 0.10 | 0.0012** | 0.0058** |
| 0.15 | 0.0084** | 0.0136* |
| 0.20 | 0.0216* | 0.0195* |
| 0.25 | 0.0225* | 0.0097** |
|  |  |  |
| *Modularity* |  |  |
| 0.05 | 0.0032** | 0.0058** |
| 0.10 | 0.0009** | 0.0039** |
| 0.15 | 0.0015** | 0.0058** |
| 0.20 | 0.0039** | 0.0097** |
| 0.25 | 0.0023** | 0.0097** |
|  |  |  |
| *Clustering coefficient* |  |  |
| 0.05 | 0.0005** | 0.0019* |
| 0.10 | 0.0002** | 0.0039* |
| 0.15 | 0.0008** | 0.0019* |
| 0.20 | 0.0023** | 0.0019* |
| 0.25 | 0.0003** | 0.0019* |
|  |  |  |

** *p* < .01 levels (*p* < .05, FWER level), * *p* < .05.
